# ERK-METTL3 axis acts as a novel regulator of antiviral innate immunity combating pseudorabies virus infection

**DOI:** 10.1371/journal.ppat.1013234

**Published:** 2025-08-13

**Authors:** Lucai Wang, Xiangqi Qiu, Lele Wang, Xilong Yang, Mengjie Li, Xuyang Zhao, Wenhui Zhu, Lijie Lv, Yunzhe Kang, Lulu Yao, Gaiping Zhang, Aijun Sun, Guoqing Zhuang

**Affiliations:** 1 International Joint Research Center of National Animal Immunology, College of Veterinary Medicine, Henan Agricultural University, Zhengzhou, China; 2 Longhu Laboratory of Advanced Immunology, Zhengzhou, China; 3 Ministry of Education Key Laboratory for Animal Pathogens and Biosafety, Zhengzhou, China; University of Wisconsin-Madison, UNITED STATES OF AMERICA

## Abstract

The ERK-mediated phosphorylation of the core m^6^A methyltransferase METTL3 has been linked to the regulation of embryonic stem cell differentiation and tumorigenesis. However, its role in the antiviral innate immune response remains unclear. In this study, we found that during infection with the prototypical alpha-herpesvirus Pseudorabies virus (PRV), ERK2 protein expression increased significantly, while METTL3 expression decreased both *in vitro* and *in vivo*. Overexpressing ERK2 and METTL3 effectively reduced PRV replication, while their knockdown promoted viral replication. The C-terminal domain and enzymatic active site of METTL3 were essential for suppressing viral replication. Mechanistically, ERK2 phosphorylates METTL3 at serine 43. We further found that ERK2-mediated phosphorylation at this site enhances the type I interferon (IFN-β)-induced innate immune response by activating the NF-κB pathway, increasing m^6^A modification, and elevating protein translation levels. Notably, combined treatment with ERK2 and METTL3 inhibitors promoted viral replication, intensified organ damage, and hastened mortality in mice by suppressing IFN-β production. In conclusion, our study reveals phosphorylation-dependent crosstalk between MAPK signaling and the m^6^A machinery in antiviral defense, identifies Ser43 as a functional hotspot for METTL3’s immunoregulatory activity, and indicates that the ERK-METTL3 axis is a novel regulator of the antiviral innate immune response during alpha-herpesvirus infection. This work establishes a paradigm shift in understanding how post-translational modifications of RNA-modifying enzymes orchestrate antiviral immunity, providing new avenues for host-directed antiviral strategies.

## Author summary

Type I interferon (IFN) is crucial for antiviral innate immunity and resistance to α-herpesvirus infections. Our findings suggest that the ERK-METTL3 axis regulates IFN production by activating NF-κB, enhancing m^6^A modification, and promoting cellular protein translation. This pathway modulates antiviral innate immunity through an amplification cascade that inhibits PRV replication. Notably, the combined inhibition of ERK2 and METTL3 significantly enhanced PRV replication *in vitro* and *in vivo*, increasing morbidity and mortality in infected mice. Thus, our study identifies the ERK-METTL3 axis as a key regulator of antiviral immune responses and a promising target for antiviral drug development.

## Introduction

Alpha-herpesviruses are a diverse group of enveloped viruses with large double-stranded DNA genomes [1]. The host’s innate immune responses to these infections are influenced not only by viral genetic material but also by covalent DNA and protein modifications. Recently, RNA modifications have emerged as important regulators of gene expression, substantially affecting host antiviral responses [2]. Among these modifications, N6-methyladenosine (m^6^A) is the most common internal mRNA modification in eukaryotes. This modification is controlled by “writer,” “eraser,” and “reader” proteins. The “writer” is a methyltransferase complex composed of Methyltransferase-like 3 (METTL3), Methyltransferase-like 14 (METTL14), Wilms tumor-associated protein (WTAP), and other co-factors [3]. METTL3, the core component, contains a zinc finger domain (ZnF) and a methyltransferase domain (MTD) that facilitates methyl transfer to specific adenine sites within the DRACH motif (D = A/G/U, R = A/G, H = U/A/C) of RNA [4,5]. This m^6^A modification can be removed by two known demethylases, fat mass and obesity-associated protein (FTO) and ALKB homologue 5 (ALKBH5) [6,7], which function as “eraser” proteins. Additionally, YTH-domain family proteins (YTHDF1, YTHDF2, YTHDF3, YTHDC1, and YTHDC2) act as “reader” by binding to and regulating the stability, splicing, translation, and degradation of m^6^A-modified RNAs [3].

Type I interferons (IFNs), such as IFN-α and IFN-β, are essential cytokines in antiviral defense [8]. The m^6^A modification of RNA regulates IFN-β responses, either enhancing or suppressing antiviral transcript expression [9–11]. METTL3, a key part of the m^6^A modification machinery, plays a multifunctional role by influencing RNA virus replication, including that of Vesicular Stomatitis Virus (VSV), through reshaping viral double-stranded RNA and inhibiting IFN-β production [12]. In contrast, TANK-binding kinase 1 (TBK1) phosphorylates METTL3, promoting IFN-β production and suppressing viral replication [13]. The extracellular signal-regulated kinases 1 and 2 (ERK1/2), downstream components of the mitogen-activated protein kinase (MAPK)-ERK pathway, regulate many biological processes by phosphorylating various substrates. These processes include cell growth, differentiation, migration, and responses to viral infections [14,15]. Recent studies indicate that ERK-mediated phosphorylation of METTL3 stabilizes the methyltransferase complex, increasing m^6^A deposition and supporting processes such as embryonic stem cell differentiation and tumorigenesis [4]. However, the antiviral role of the ERK-METTL3 axis remains unexplored.

Pseudorabies virus (PRV), an *Alphaherpesvirinae* subfamily member, infects multiple mammals, in very rare cases, may possibly infect humans [16]. This double-stranded DNA virus has a genome with unique long (UL) and short (US) regions flanked by terminal (TR) and internal (IRL) repeat sequences [17]. PRV has evolved several strategies to evade host immunity. As a prototypical alpha-herpesvirus, PRV-infected hosts serve as a valuable model for studying how epitranscriptomic modifications affect antiviral innate immunity in alpha-herpesviruses.

In this study, we show that the ERK-METTL3 axis enhances antiviral innate immunity through ERK2-mediated phosphorylation of METTL3. This activation promotes NF-κB, increases m^6^A modification, and elevates protein translation levels. Notably, inhibiting both ERK2 and METTL3 together increased viral replication and accelerated PRV pathogenesis. Overall, our findings provide insight into the antiviral functions of the ERK-METTL3 axis in alpha-herpesviruses, offering new perspectives for potential therapeutic strategies.

## Results

### PRV infection notably increased ERK1/2 expression while reducing METTL3 expression both *in vitro* and *in vivo*

**Fig 1:**
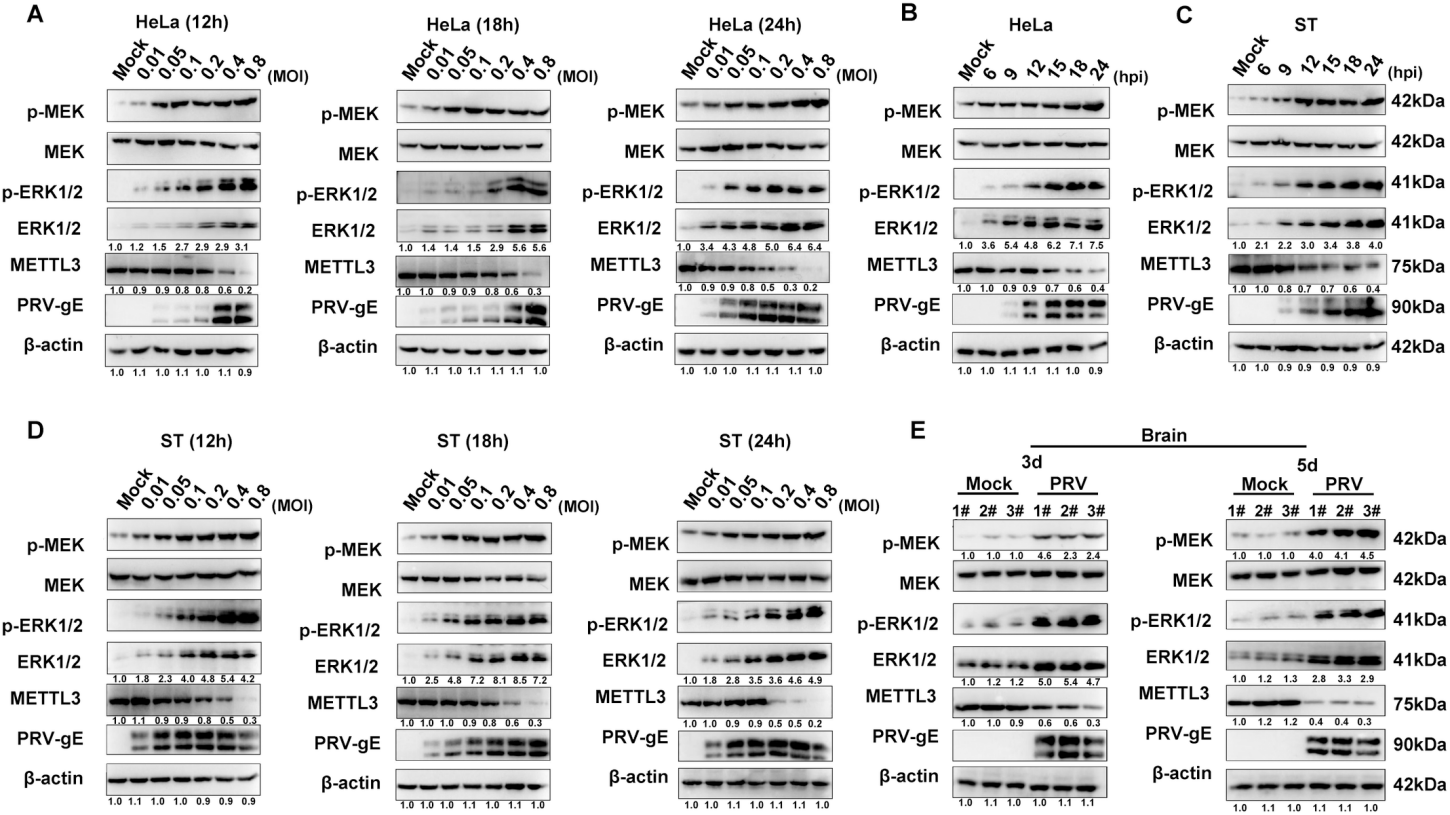
The altered expression patterns of MAPK signaling pathway-related proteins and METTL3 during PRV infection. (A) HeLa cells were infected with PRV at multiplicities of infection (MOI = 0.01, 0.05, 0.1, 0.2, 0.4, 0.8) at different time points (12, 18, and 24 hpi). The expression levels of MAPK signaling pathway-related proteins and METTL3 were assessed using Western blot. p-ERK1/2: Phosphorylated ERK1/2, MEK: Mitogen-Activated Protein Kinase Kinase, p-MEK: Phosphorylated MEK. **(B)** HeLa cells were infected with PRV (MOI = 0.4), ST cells were collected at different time points (0, 6, 9, 12, 15, 18, and 24 h), and the expression of MAPK signaling pathway-related proteins and METTL3 was assessed by Western blot. **(C)** ST cells were collected at various time points (0, 6, 9, 12, 15, 18, and 24 h) after infection with PRV (MOI = 0.4), and the expression of MAPK signaling pathway-related proteins and METTL3 was detected by Western blot.** (D)** ST cells were infected with PRV at multiplicities of infection (MOI = 0.01, 0.05, 0.1, 0.2, 0.4, 0.8) at different time points (12, 18, and 24 hpi). The expression levels of MAPK signaling pathway-related proteins and METTL3 were assessed using Western blot. (E) Western blot analysis was performed to detect the activation of MAPK signaling pathway-related proteins and METTL3 in the brains of mice infected with PRV at 3 or 5 dpi. The mock group comprised uninfected and untransformed samples.

To examine how PRV infection affects ERK1/2 and METTL3 expression *in vitro*, HeLa cells were infected with PRV at multiplicities of infection (MOI = 0.01, 0.05, 0.1, 0.2, 0.4, 0.8). Western blot analysis revealed a significant, dose-dependent increase in phosphorylated MEK, ERK1/2, and phosphorylated ERK1/2 expression at 12, 18 and 24 h post-infection (hpi) (Fig 1A). Next, HeLa cells were infected with PRV (MOI = 0.4), and samples were collected at 6, 9, 12, 15, 18, and 24 hpi. This analysis showed a significant, time-dependent increase in phosphorylated MEK, ERK1/2, and phosphorylated ERK1/2 expression (Fig 1B). We examined the temporal-spatial distribution of ERK1/2 following PRV infection and observed a gradual translocation from the cytoplasm to the nucleus over time, indicating ERK1/2 activation (S1A Fig). Concurrently, Western blot indicated that METTL3 expression was significantly downregulated in a time- and dose-dependent manner after viral infection (Fig 1A and 1B). These findings were confirmed in PRV-infected ST cells, ruling out cell-specific effects (Fig 1C and 1D).

We then investigated whether the PRV-induced expression patterns of ERK1/2 and METTL3 observed *in vitro* were also present *in vivo*. For this, mice were infected with PRV (10^5^TCID_50_ per mouse) in the experimental group, while the control group received PBS injections only. Brain samples were collected at three- and five- day post-infection (dpi) for Western blot. Compared to the PBS control group, the PRV-infected group showed significantly higher expression of phosphorylated MEK, ERK1/2, and phosphorylated ERK1/2, while METTL3 expression was significantly downregulated (Fig 1E). Interestingly, similar expression patterns were observed in spleen samples, indicating no tissue-specific differences in these proteins’ expression (S1B Fig). In summary, these findings demonstrate that PRV infection activates ERK1/2 and suppresses METTL3 expression both *in vitro* and *in vivo*.

### ERK-METTL3 axis significantly inhibits PRV replication

**Fig 2:**
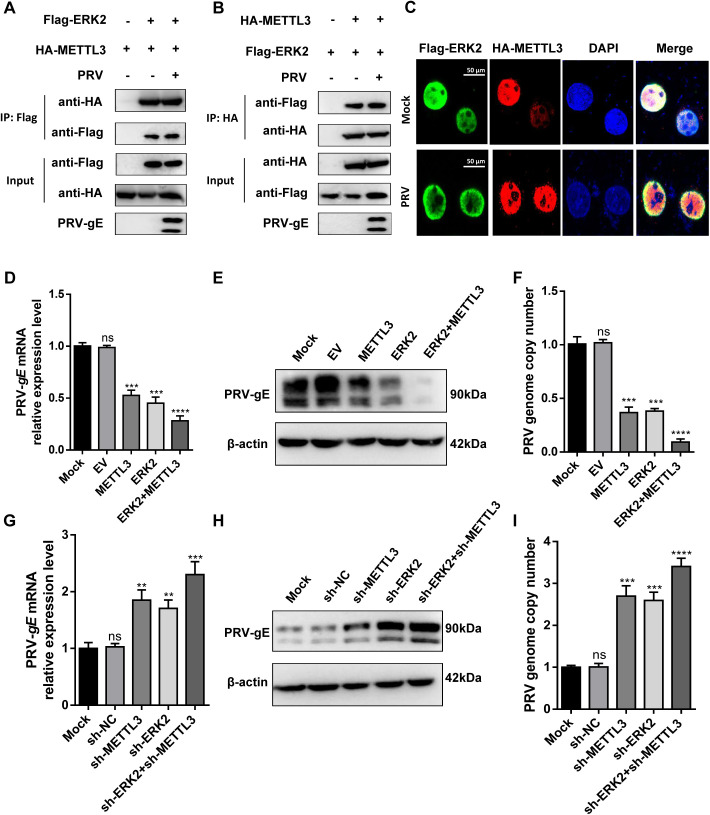
ERK2 interacts with METTL3 to inhibit PRV replication. Flag beads **(A)** or HA beads** (B)** were used to detect the interaction between ERK2 and METTL3 in both PRV-infected (MOI = 0.4) and uninfected cells. (**C)** HeLa cells were co-transfected with 3 × Flag-CMV-14-ERK2 and pCAGGS-HA-METTL3. After 12 h, cells were infected with PRV (MOI = 0.4), with non-inoculated controls and empty vectors included. Samples were processed 24 h post-infection, and laser confocal microscopy was used to verify the co-localization of ERK2 and METTL3. HeLa cells were transfected with empty vectors, pCAGGS-HA-METTL3, 3 × Flag-CMV-14-ERK2, or a combination of pCAGGS-HA-METTL3 and 3 × Flag-CMV-14-ERK2. After 12 h, the cells were infected with PRV (MOI = 0.4). Samples were collected 24 h later, and *gE* mRNA expression was analyzed by qPCR **(D)**, while gE protein expression was assessed by Western blot **(E)**. Changes in viral DNA copy number were quantified using qPCR **(F)**. HeLa cells were transfected with an empty vector, sh-METTL3, sh-ERK2, or a combination of sh-METTL3 and sh-ERK2. After 12 h, the cells were infected with PRV (MOI = 0.4). Samples were collected 24 h later, and *gE* mRNA expression was analyzed by qPCR **(G)**, while gE protein expression was assessed by Western blot **(H)**. Changes in viral DNA copy number were quantified using qPCR **(I)**. The final results are presented as the normalized viral DNA copy number, with error bars representing the standard error of the mean. Data were shown as mean ± SD based on three independent experiments. ** p < 0.01, *** p < 0.001, **** p < 0.0001 determined by two-tailed Student’s t-test. The mock group in Fig 2B was not infected with PRV but transfected with the plasmid containing Flag-ERK2 and HA-METTL3. The mock group in Fig 2D-2I was the untransfected group infected with PRV. EV: Empty vector control, which contains no target sequence. sh-NC: shRNA empty vector control, which contains the shRNA scaffold but no specific target sequence.

A previous study reported that ERK1/2 interacts with and phosphorylates METTL3, forming an axis that regulates stem cell differentiation and tumorigenesis [4]. We initially confirmed this interaction and phosphorylation during PRV infection. Due to the high similarity and identical substrate specificity of ERK1 and ERK2 *in vitro*, and since ERK2 is more abundantly expressed than ERK1 in most cells, we selected ERK2 for subsequent experiments [4]. Co-immunoprecipitation (Co-IP) analysis confirmed the interaction between ERK2 and METTL3, which persisted during PRV infection, demonstrating that ERK2 interacts with METTL3 independently of PRV infection (Fig 2A and 2B). Using confocal laser microscopy, we further investigated the impact of PRV infection on the colocalization of ERK2 and METTL3 within cells. Our findings revealed that, in the absence of PRV infection, ERK2 and METTL3 are colocalized within the nucleus, a result consistent with previous report [4]. Notably, even after PRV infection, ERK2 and METTL3 remained present in the nucleus (Fig 2C). Additionally, we analyzed the distribution of endogenous ERK1/2 in PRV-infected and non-infected cells. PRV infection triggered ERK2 activation, driving its translocation to the nucleus (S2A Fig). We propose that in the absence of PRV infection, the nuclear colocalization of ERK2 and METTL3 may result from METTL3’s nuclear localization signal (NLS) or interaction domain, which facilitates its nuclear recruitment. To explore the role of ERK2 and METTL3 in viral replication, we constructed 3 × Flag-cmv-14-ERK2 and pCAGGS-HA-METTL3 overexpression plasmids and transfected them, along with empty vectors, into HeLa cells. After 12 h, cells were infected with PRV (MOI = 0.4), and samples were collected at 24 hpi. The results showed that overexpression of ERK2 or METTL3 significantly downregulated PRV replication genes *gE* and *gB* mRNA (Figs 2D and S2B) and reduced gE protein levels (Fig 2E). Notably, co-transfection of ERK2 and METTL3 led to a significant reduction in viral DNA copy number (Fig 2F), suggesting that ERK2 and METTL3 overexpression inhibits PRV replication. Next, we assessed PRV replication following ERK2 and METTL3 depletion. We generated ERK2 and METTL3 knockout cell lines, validated by qPCR and Western blot (S3A–S3D Fig). In these knockout cell lines, PRV-*gE* and *gB* mRNA levels, as well as gE protein levels and viral DNA copy number, were significantly upregulated (S4A–S4D Fig), indicating that ERK2 or METTL3 knockout promotes PRV replication. To further investigate their roles, we generated ERK2 and METTL3 knockout PK-15 cell lines using sgRNA and confirmed knockout efficiency (S5A–S5D Fig). The results were consistent in HeLa cells. In ERK2- and METTL3-knockout PK-15 cells, PRV-*gE* and *gB* mRNA levels, gE protein levels, and viral DNA copy number were up-regulated (S6A-S6D Fig). However, full and double knockout significantly reduced cell viability, prompting us to use shRNA for further experiments. We constructed shRNAs targeting ERK2 and METTL3 and found that sh-ERK2 or sh-METTL3 significantly upregulated PRV-*gE* and *gB* mRNA expression (Figs 2G and S2C), as well as gE protein levels (Fig 2H). Furthermore, the viral DNA copy number significantly increased post-transfection with sh-ERK2 or sh-METTL3. Notably, co-transfection with both sh-ERK2 and sh-METTL3 caused an additional increase in PRV-gE protein and viral DNA copy number compared to individual shRNAs (Fig 2I). These findings demonstrate that ERK2 and METTL3 deficiency promotes PRV replication.

### The METTL3 enzyme activity integrity is important for the ERK-METTL3 axis functionality

**Fig 3:**
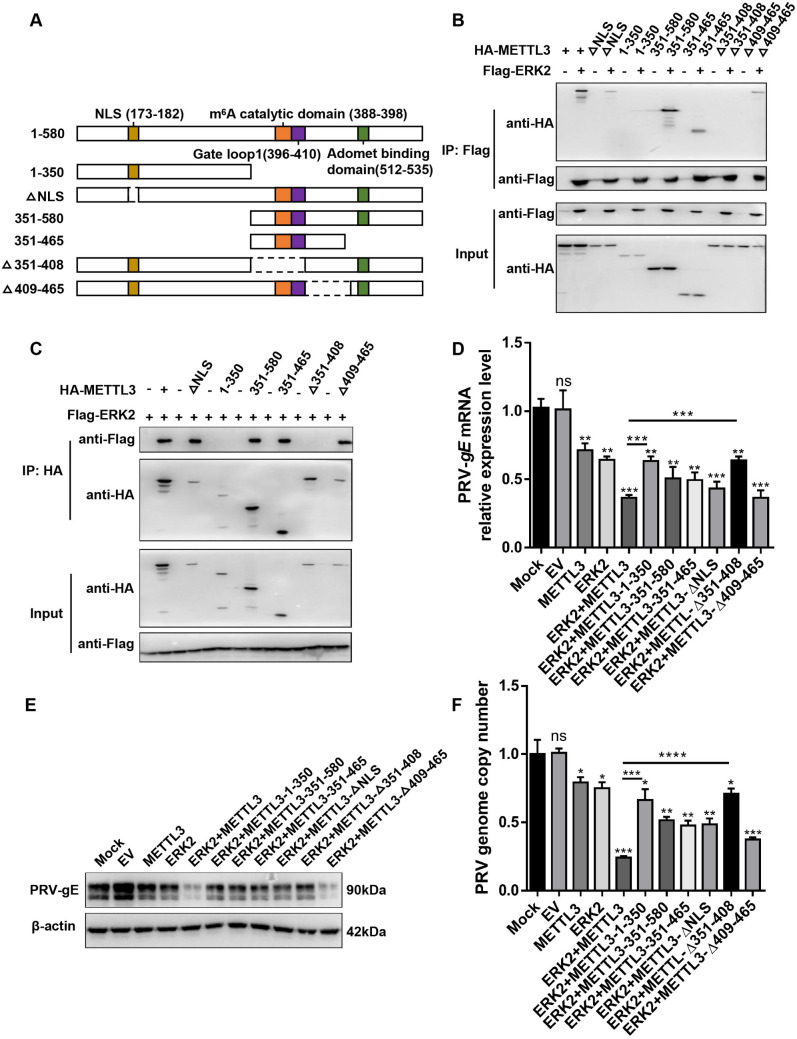
The C-terminal domain of METTL3 (aa) interacts with ERK2 and is essential for PRV replication. **(A)** Schematic representation of METTL3 truncations and mutants. The interaction between ERK2 and the METTL3 truncations was assessed using Flag beads **(B)** or HA beads **(C).** The 3 × Flag-CMV-14-ERK2 was co-transfected with pCAGGS-HA-METTL3, its mutant, and truncate respectively, and overexpressed with 3 × FLag-CMV-14-ERK2 and pCAGGS-HA-METTL3, respectively, along with appropriate no-load and blank controls. After 12 h post-transfection, PRV (MOI = 0.4) was introduced, and samples were collected 24 h after infection. The expression of *gE* mRNA** (D)** and protein **(E)** was analyzed using qPCR and Western blot, respectively.** (F)** qPCR was employed to quantify the viral DNA copy number. The final results are presented as the normalized viral DNA copy number, with error bars representing the standard error of the mean. Data were shown as mean ± SD based on three independent experiments. * p < 0.05, ** p < 0.01, *** p < 0.001, **** p < 0.0001 determined by two-tailed Student’s *t*-test. EV: Empty vector control, which contains no target sequence. The mock group in Fig 3D-3F is the group that was not transfected but infected with PRV.

This study investigates the structural basis of the ERK-METTL3 interaction and its antiviral mechanism. We generated a series of truncated mutants (Fig 3A), and Co-IP analysis showed that ERK2 did not interact with METTL3-∆351–408, indicating that ERK2 specifically binds to METTL3 residues 351–408. Previous studies have identified aspartic acid at position 395 within the m^6^A catalytic core (388–398) as critical for METTL3 activity [18]. Based on this, we propose that ERK2 interacts with METTL3’s catalytically active region (Fig 3B and 3C). Co-transfection of ERK2 with METTL3-∆351–408 significantly reduced PRV-*gE* mRNA inhibition, protein expression (Fig 3D and 3E), and viral DNA copy number (Fig 3F) in PRV-infected HeLa cell models, highlighting this region’s essential role in antiviral function. Notably, a fragment comprising residues 1–350, which lacks ERK2-binding capacity, still exhibited weak antiviral activity, suggesting an ERK2-independent defense mechanism. Further analysis showed that truncates 351–580 and 351–465, which retain the catalytic domain, partially inhibited viral replication, albeit less effectively than full-length METTL3. Mechanistically, NLS loss may lead to aberrant subcellular localization, while deletion of the 43 phosphorylation site may impair METTL3 activity, disrupting complex function. Interestingly, the ∆409–465 mutant, which retains both the catalytic domain and NLS, exhibited antiviral activity comparable to the full-length protein (Fig 3F), suggesting functional redundancy in this region. Collectively, METTL3 forms a dynamic interaction network with ERK2 via its catalytic domain, potentially enhancing antiviral effects by stabilizing enzyme conformation or modulating substrate specificity. These findings suggest that the ERK–METTL3 axis’s antiviral function depends on the integrity of METTL3 enzymatic activity.

### The phosphorylation of METTL3 at the S43 site by ERK2 is essential for its effect on viral replication

**Fig 4:**
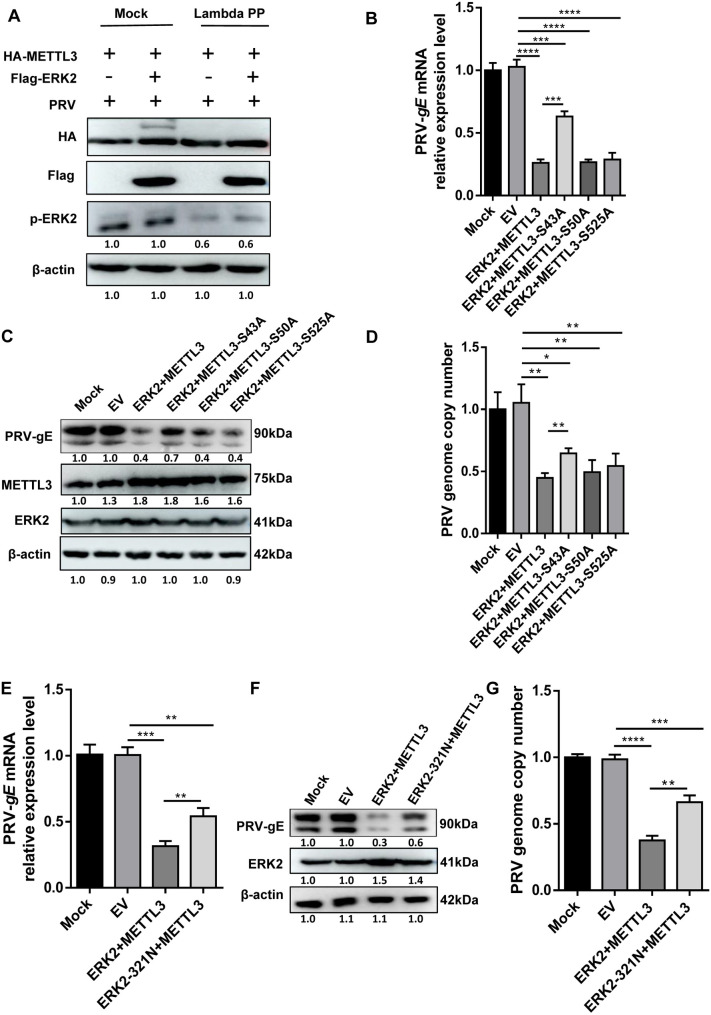
The METTL3 phosphorylation sites targeted by ERK2 are essential for PRV replication. (A) HeLa cells were co-transfected with pCAGGS-HA-METTL3 and either 3 × Flag-CMV-14-ERK2 or an empty vector for 24 h. Whole-cell lysates were either mock-treated or treated with Lambda protein phosphatase (Lambda PP) just before SDS-PAGE. Following this, 3 × Flag-CMV-14-ERK2 was co-transfected with pCAGGS-HA-METTL3, pCAGGS-HA-S43A, pCAGGS-HA-S50A, and pCAGGS-HA-S525A. The cells were then infected with PRV (MOI = 0.4) 12 h later. Cell samples were collected 24 h post-infection, and *gE* mRNA expression **(B)** was measured using qPCR. The expression of gE protein **(C**) and the viral DNA copy number **(D**) were assessed via Western blot and qPCR. Additionally, pCAGGS-HA-METTL3 was co-transfected with 3 × FLag-CMV-14-ERK2 and 3 × FLag-CMV-14-ERK2-321N. After 12 h, cells were infected with PRV (MOI = 0.4), and 24 h later, *gE* mRNA expression **(E)** was measured by qPCR. The expression of gE protein **(F)** and the viral DNA copy number **(G)** were determined using Western blot and qPCR. The final results are presented as the normalized viral DNA copy number, with error bars representing the standard error of the mean. Data were shown as mean ± SD based on three independent experiments. * p < 0.05, ** p < 0.01, *** p < 0.001 **** p < 0.0001 determined by two-tailed Student’s *t*-test. EV: Empty vector control, which contains no target sequence. The mock group in Fig 4B-4G was not transfected but infected with PRV.

ERK1/2 is a serine/threonine kinase. Previous studies have shown that ERK phosphorylates METTL3 at positions S43, S50, and S525 [4]. We experimentally confirmed that ERK2 phosphorylates METTL3 (Fig 4A). To investigate the role of these phosphorylation sites in virus replication, we individually mutated each site and co-transfected them with ERK2 into HeLa cells. We measured *gE* mRNA expression, protein levels, and viral DNA copy number to assess the impact of phosphorylation on viral replication. Co-transfection of ERK2 and METTL3 significantly inhibited viral replication. However, the inhibitory effect was reduced when co-transfected with a mutant plasmid containing the S43A phosphorylation site mutation, as observed in decreased PRV-*gE* and *gB* mRNA expression (Figs 4B and S7), and gE protein levels (Fig 4C). Mutations at the S50A and S525A phosphorylation sites did not significantly differ from wild-type (WT) METTL3 in terms of their inhibitory effects (Fig 4C and 4D). Viral DNA copy number assays revealed that the S43A phosphorylation site mutation led to a significant increase in viral copy number compared to WT METTL3 (Fig 4D). Collectively, these demonstrate that the S43A phosphorylation site mutation significantly reduces the inhibitory effect on viral replication. The ERK2-321N mutation disrupts the interaction between ERK2 and METTL3 [4]. To assess whether this interaction is altered during PRV infection and its impact on viral replication, we co-transfected METTL3 with ERK2-321N. Co-transfection partially restored *gE* and *gB* mRNA expression, gE protein levels, and viral DNA copy number (Figs 4E, 4F, and S8). Viral DNA copy number assays showed that co-transfection of METTL3 and ERK2-321N resulted in a significant reduction in viral inhibition compared to co-transfection of METTL3 and WT ERK2 (Fig 4G). These findings indicate that the loss of interaction between ERK2 and METTL3 results in a significant reduction in the enhanced inhibition of viral replication. In conclusion, the phosphorylation of METTL3 at the S43 site by ERK2 is a crucial component in inhibiting viral replication.

### ERK-METTL3 axis facilitates antiviral innate immune responses during PRV infection

**Fig 5:**
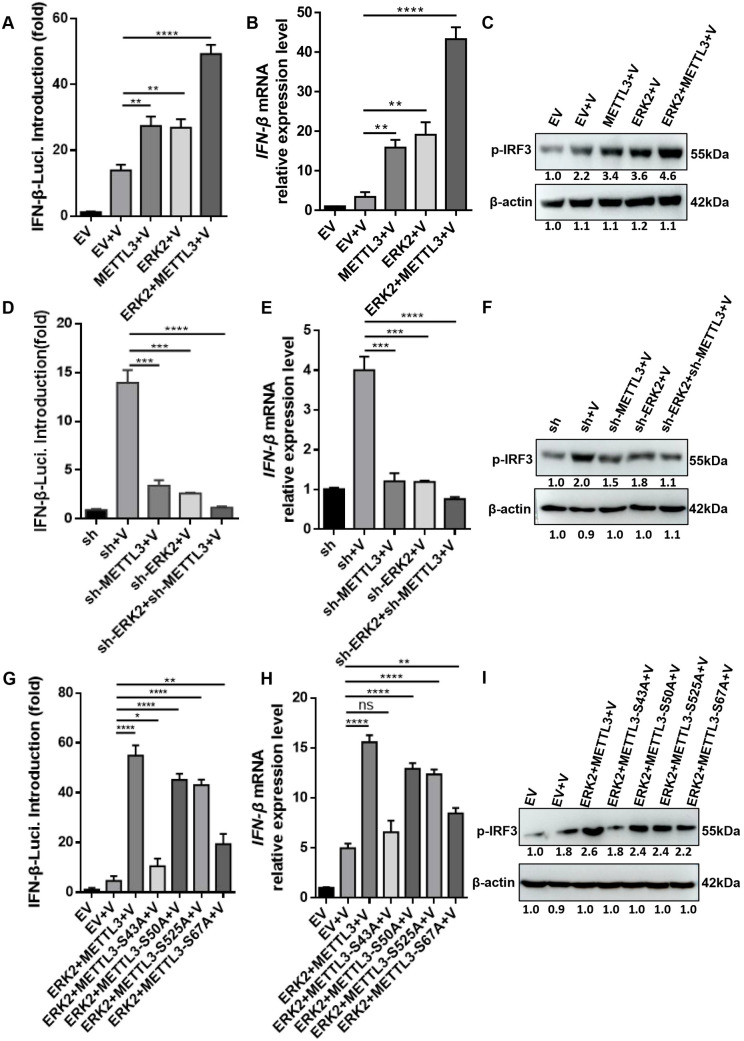
ERK2 and METTL3 interact to affect interferon-beta production during PRV replication. (A) HeLa cells were co-transfected with 3 × FLag-CMV-14-ERK2 (200 ng), pCAGGS-HA-METTL3 (200 ng), or combinations of 3 × FLag-CMV-14-ERK2 (100 ng) with pCAGGS-HA-METTL3 (100 ng) along with IFN-β luciferase reporter plasmids (125 ng) and TK (25 ng). After 12 h, the cells were infected with PRV (MOI = 0.4), and a blank control was established. After 24 h post-infection, cell samples were collected for a dual luciferase assay. (B) HeLa cells were transfected with empty vector, 3 × FLag-CMV-14-ERK2, pCAGGS-HA-METTL3, and a combination of 3 × FLag-CMV-14-ERK2 + pCAGGS-HA-METTL3, then infected with PRV (MOI = 0.4) 12 h later. A blank control group was also established. Cell samples were collected 24 h later for qPCR detection of *IFN-β* mRNA expression. (C) The same treated cells as in (B) were analyzed for p-IRF3 expression by Western blot. (D) HeLa cells were co-transfected with sh-ERK2 (200 ng), sh-METTL3 (200 ng), or combinations of sh-ERK2 (100 ng) with sh-METTL3 (100 ng), along with IFN-β luciferase reporter plasmids (125 ng) and TK (25 ng). After 12 h, the cells were infected with PRV (MOI = 0.4), and a blank control was established. After 24 h post-infection, cell samples were collected for a dual luciferase assay. (E) HeLa cells were transfected with empty vectors, sh-ERK2, sh-METTL3, and a combination of sh-ERK2 + sh-METTL3, and infected with PRV (MOI = 0.4) 12 h later, with a blank control group established. Cell samples were collected 24 h later for qPCR detection of *IFN-β* mRNA expression. (F) The same treated cells as in (E) were analyzed for p-IRF3 expression by Western blot. (G) HeLa cells were co-transfected with 3 × FLag-CMV-14-ERK2 (100 ng) and pCAGGS-HA-METTL3 (100 ng), pCAGGS-HA-S43A (100 ng), pCAGGS-HA-S50A (100 ng), pCAGGS-HA-S525A (100 ng), or pCAGGS-HA-S67A (100 ng), along with IFN-β luciferase reporter plasmids (125 ng) and TK (25 ng). After 12 h, the cells were infected with PRV (MOI = 0.4), and a blank control was established. At 24 h post-infection, cell samples were collected for a dual luciferase assay. **(H)** HeLa cells were co-transfected with 3 × FLAG-CMV-14-ERK2 and pCAGGS-HA-METTL3, pCAGGS-HA-S43A, pCAGGS-HA-S50A, pCAGGS-HA-S525A or pCAGGS-HA-S67A, and then infected with PRV (MOI = 0.4) 12 h later. Cell samples were collected 24 h later for qRT-PCR detection of *IFN-β* mRNA expression. **(I)** The same treated cells as in **(H)** were analyzed 24 h later for p-IRF3 protein expression. Data were shown as mean ± SD based on three independent experiments. * p < 0.05, ** p < 0.01, *** p < 0.001 **** p < 0.0001 determined by two-tailed Student’s *t*-test. EV: Empty vector control, which contains no target sequence. sh: shRNA empty vector control, which contains the shRNA scaffold but no specific target sequence. V: Samples infected with PRV.

Type I interferon (IFN) plays a significant role in combating virus infection [19]. We investigated whether ERK2 and METTL3 influence viral replication through the regulation of IFN production. To elucidate the interaction between ERK2 and METTL3’s influence on IFN-β production, we conducted a series of experiments using a dual-luciferase reporter assay and qPCR. HeLa cells were co-transfected with an IFN-β luciferase reporter plasmid and TK, along with either the ERK2 or METTL3 overexpression plasmids. Following infection with PRV (MOI = 0.4), IFN-β promoter activity was measured. The results showed that ERK2 and METTL3 overexpression can individually activate the IFN-β promoter activity. When both ERK2 and METTL3 are overexpressed, the activation level is significantly increased (Fig 5A). To validate these findings, qRT-PCR was used to detect the mRNA levels of *IFN-β*, which confirmed the results from the dual-luciferase reporter assay. When ERK2 and METTL3 act together, the mRNA level of *IFN-β* is significantly elevated (Fig 5B). Interferon regulatory factor 3 (IRF3) is a key transcription factor in innate immune responses, and its activation state is crucial for the expression of IFN-β. We hypothesized that the regulation of IFN-β occurs through the activation of IRF3. Therefore, we examined the expression of p-IRF3 protein. Interestingly, transfection with ERK2 or METTL3 upregulates the expression of p-IRF3, and their combined action substantially increases the protein expression of p-IRF3 (Fig 5C). These findings were validated using shRNAs. Through the dual-luciferase reporter assay, we confirmed that both sh-ERK2 and sh-METTL3 can inhibit the activity of the IFN-β promoter, and their combined effect leads to a significant inhibition of IFN-β activity (Fig 5D). qRT-PCR results were consistent with those from the dual-luciferase reporter assay, sh-ERK2 and sh-METTL3 significantly suppress the expression of *IFN-β* mRNA (Fig 5E). The activation status of IRF3 was also verified, and as expected, sh-ERK2 and sh-METTL3 substantially downregulated the protein expression of p-IRF3 (Fig 5F). Overall, our findings demonstrate that in the context of PRV infection, the ERK-METTL3 complex regulates the induction of IFN production by activating IRF3.

Further, we investigated the role of METTL3 phosphorylation sites in IFN-β production using METTL3 phosphorylation site mutants (S43A, S50A, S67A, and S525A) and employed a dual-luciferase reporter assay to measure IFN-β activation. We also conducted qPCR to evaluate *IFN-β* mRNA expression and Western blot to examine p-IRF3 protein levels. Our findings revealed that the mutation of the S43 phosphorylation site significantly diminished IFN-β activation (Fig 5G), as evidenced by reduced *IFN-β* mRNA levels and lower p-IRF3 protein levels (Fig 5H and 5I). In conclusion, during PRV infection, the ERK-METTL3 axis positively influences IFN production, and the S43 phosphorylation site plays a crucial role in this regulatory mechanism.

### ERK2 interacts with and phosphorylates METTL3 promoted interferon-beta production through activating NF-κB signaling during PRV infection

**Fig 6:**
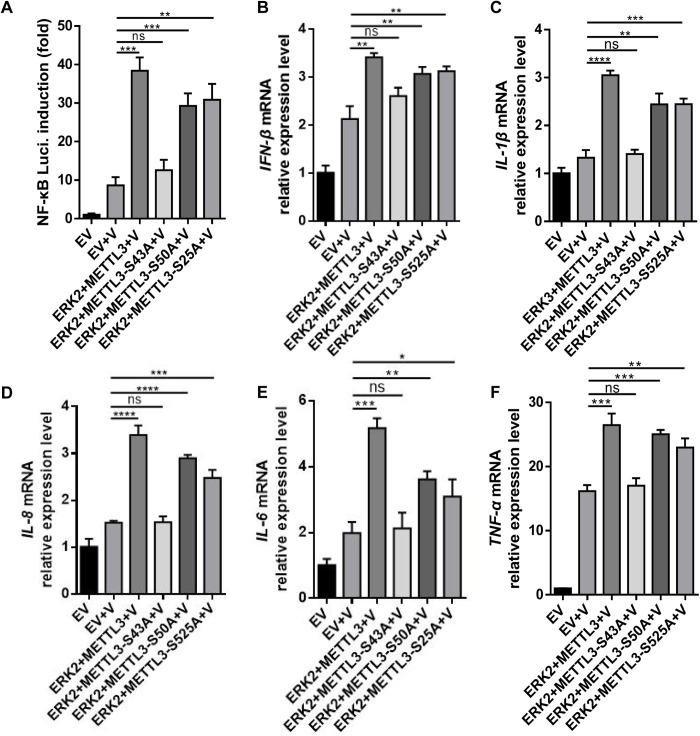
ERK2 interacts with and phosphorylates METTL3, promoting interferon-beta production by activating NF-kB signaling during PRV infection. (A) HEK-293T cells were transfected with pGL4.17-NF-κB-Luc and PRL-TK, along with empty vector, pCMV-3xFlag-ERK2, pCAGGS-HA-METTL3, pCAGGS-HA-METTL3-S43A, pCAGGS-HA-METTL3-S50A, and pCAGGS-HA-METTL3-S525A for 18 h. Following transfection, the cells were infected with PRV (MOI = 0.4) for an additional 18 h. The cells were then collected to measure luciferase activity. (B) HEK-293T cells were transfected with empty vector, NF-κB plasmid, pCMV-3xFlag-ERK2, and pCAGGS-HA-METTL3 for 18 h, followed by infection with PRV (MOI = 0.4) for another 18 h. The cells were then collected to assess the mRNA levels of *IFN-β*. Additionally, HeLa cells were transfected with empty vector, pCMV-3xFlag-ERK2, pCAGGS-HA-METTL3, pCAGGS-HA-METTL3-S43A, pCAGGS-HA-METTL3-S50A, and pCAGGS-HA-METTL3-S525A for 18 h, followed by infection with PRV (MOI = 0.4) for another 18 h. These cells were then collected to measure the mRNA levels of *IL-1β* (C), *IL-8* (D), *IL-6* (F) and *TNF-α* (F) using qPCR. Data were shown as mean ± SD based on three independent experiments. * p < 0.05, ** p < 0.01, *** p < 0.001, **** p < 0.0001 determined by two-tailed Student’s *t*-test. EV: Empty vec*t*or control, which contains no target sequence. V: Samples infected with PRV.

Previous studies have shown that METTL3 redistribution can bind to the NF-κB promoter [20]. We proposed that ERK2-mediated phosphorylation of METTL3 might also influence the NF-κB signaling pathway during PRV infection. To explore this, we examined the effects of different ERK2 and METTL3 site mutants (S43A, S50A, and S525A) on NF-κB promoter activity. Our results revealed that all METTL3 groups (METTL3, METTL3-S43A, METTL3-S50A, and METTL3-S525A) significantly enhanced NF-κB promoter activity during PRV (MOI = 0.4) infection compared to the empty vector group. However, the METTL3-S43A group exhibited a reduced enhancement effect compared to the METTL3-S50A and METTL3-S525A groups (Fig 6A). NF-κB activation is a key factor in IFN induction [21]. We co-transfected NF-κB, ERK2, and METTL3 plasmids and established empty vector controls. We observed a significant increase in *IFN-β* mRNA expression when all three were co-transfected. The METTL3-S43A group significantly reduced this enhancement effect compared to the METTL3-S50A and METTL3-S525A groups (Fig 6B). NF-κB activation leads to the production of various cytokines [22]. To further investigate whether ERK2-mediated METTL3 phosphorylation is involved in endogenous NF-κB signaling, we analyzed the transcription levels of several inflammatory factors in HeLa cells overexpressing different site mutants of METTL3 (S43A, S50A, and S525A) after PRV infection using qRT-PCR. The results showed that, during PRV (MOI = 0.4) infection, compared to the empty vector group, all METTL3 groups (METTL3, METTL3-S43A, METTL3-S50A, and METTL3-S525A) significantly enhanced the mRNA transcription levels of *IL-1β* (Fig 6C), *IL-8* (Fig 6D), *IL-6* (Fig 6E) and *TNF-α* (Fig 6F). However, the METTL3-S43A group exhibited a significantly reduced enhancement effect compared to the METTL3-S50A and METTL3-S525A groups. Therefore, these findings strongly support the hypothesis that ERK2 phosphorylates METTL3 to activate the NF-κB signaling pathway, thereby regulating IFN production. Additionally, the phosphorylation site of S43 is crucial for METTL3 to activate the NF-κB pathway and regulate IFN production.

### ERK2 and METTL3 interaction is required for enhanced m^6^A modification and promoted cellular protein translation during PRV infection

**Fig 7:**
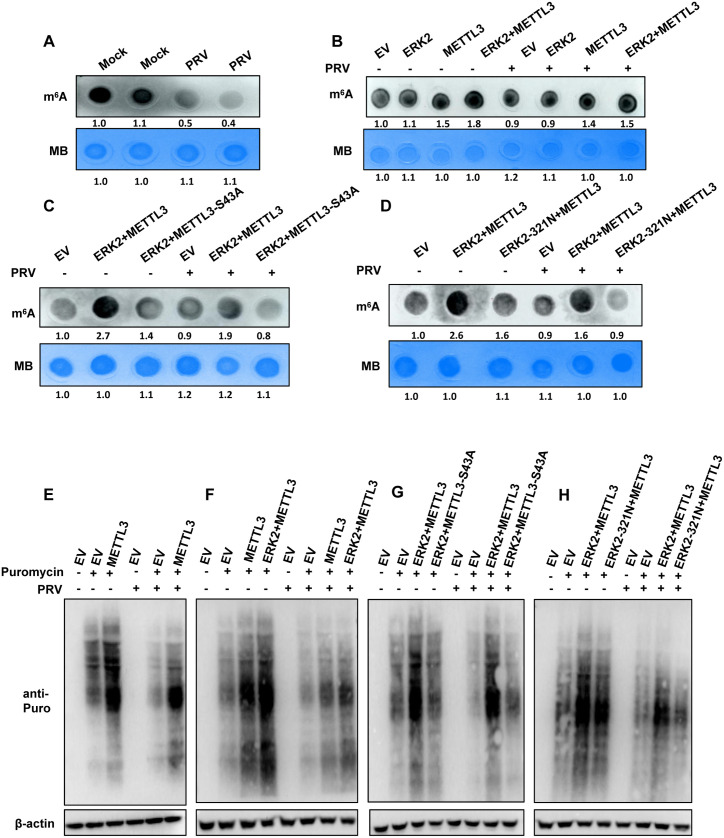
ERK2 induces phosphorylation of METTL3, enhancing m^6^A modification and promoting protein translation during viral infection. (A) HeLa cells were divided into two groups: one infected with PRV (MOI = 0.4) and one untreated. RNA was extracted for the m^6^A dot assay after 12 h. (B) The indicated plasmids were transfected into HeLa cells; 24 h later, one group was infected with PRV (MOI = 0.4), while the other remained untreated. RNA was extracted 12 h post-infection for the m^6^A dot assay. (C) The m^6^A dot assay was conducted to evaluate the impact of S43A on cellular m^6^A levels. (D) The m^6^A dot assay was utilized to assess the effect of ERK2-321N on cellular m^6^A levels. (E) Plasmids were transfected separately into HeLa cells. One group was infected with PRV (MOI = 0.4) for 12 h, while the other was left uninfected. The cells were treated with puromycin (1 μg/mL) 30 min before sample collection. (F) Puromycin treatment was employed to investigate the role of the ERK-METTL3 axis in cellular protein translation. (G) The puromycin treatment methodology was used to determine the effect of S43 of METTL3 on cellular protein translation. (H) The impact of ERK2 kinase activity on cellular protein translation was assessed using the puromycin treatment methodology. EV: Empty vector control, which contains no target sequence. MB: Methylene Blue is commonly used to standardize RNA input amounts in studies of m^6^A modification.

Previous studies have shown that RNA m^6^A modification levels may increase or decrease during viral infection [23,24]. Recent research reveals that TBK1 phosphorylates and interacts with METTL3, raising m^6^A modification levels. Importantly, the S67A mutant of METTL3 retains RNA m^6^A methylation activity similar to that of wild-type METTL3. Increased m^6^A modification has been found to promote IRF3 expression, which then induces IFN production [13]. We found that the previously found ERK2-meditated phosphorylation of METTL3 at the S43 site activated IFN-β expression in our study (Fig 5G and 5H). We aimed to examine if the ERK-METTL3 axis affects IFN-β expression by regulating cellular m^6^A levels. Initially, we observed that PRV infection reduces cellular m^6^A levels, which aligned with previous studies [24] (Fig 7A). However, METTL3 overexpression increased m^6^A modification levels. To explore whether ERK2 enhances METTL3-mediated m^6^A modification, we measured m^6^A levels catalyzed by METTL3 in the presence of ERK2. As expected, although PRV infection globally reduced m^6^A levels, the overexpression of both ERK2 and METTL3 upregulated m^6^A modification levels irrespective of PRV infection status (Fig 7B). We next investigated the role of the S43 phosphorylation site in METTL3’s catalytic activity. Interestingly, the S43A mutant of METTL3 reduced m^6^A modification levels, regardless of PRV infection (Fig 7C). Similarly, the ERK2-321N mutant reduced cellular m^6^A levels irrespective of PRV infection (Fig 7D). These findings suggest that the S43 phosphorylation site is essential for m^6^A modification in ERK2-mediated phosphorylation of METTL3. In conclusion, ERK2-mediated METTL3 phosphorylation enhances m^6^A modification, with or without the PRV infection.

Previous studies have shown that TBK1 strengthens the interaction between METTL3 and translation-related proteins. This interaction is nearly eliminated when the S67 site of METTL3 is mutated [13]. Our study finds that METTL3 can boost cellular protein translation even without viral infection. Remarkably, during PRV infection, although overall translation levels decrease compared to uninfected cells, METTL3 continues to enhance protein translation (Fig 7E). To investigate ERK2’s regulatory role in the interaction between METTL3 and translation-related proteins, we co-transfected ERK2 and METTL3. Our results show that ERK2 substantially promotes this interaction with or without PRV infection (Fig 7F). This suggests that ERK2’s phosphorylation of METTL3 enhances protein translation levels during PRV infection. We also examined the effect of METTL3 S43 phosphorylation on translation levels. We found that mutating the S43 site substantially reduces translation during PRV infection (Fig 7G). Therefore, we propose that ERK2 may enhance translation levels by phosphorylating METTL3 at the S43 site. Furthermore, we observed that the ERK2-321N mutant, unlike WT ERK2, reduces translation levels when co-expressed with METTL3 during PRV infection (Fig 7H). In summary, these findings suggest that despite the overall reduction in translation levels due to PRV infection, the ERK-METTL3 axis can still promote translation. This is likely mediated through ERK2-induced phosphorylation of METTL3 at the S43 site.

### The inhibitors of ERK2 and METTL3 significantly promote PRV replication *in vitro*

**Fig 8:**
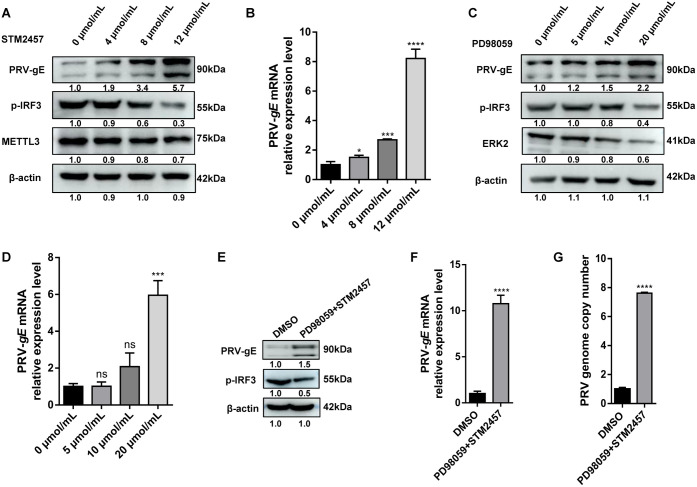
STM2457 and PD98059 promoted PRV replication *in vitro.* (A) Western blot was performed to analyze the protein levels of gE and phosphorylated IRF3 (p-IRF3) in HeLa cells treated with various concentrations of STM2457 for 24 h, followed by PRV infection for 18 h. (B) qPCR analysis of PRV-*gE* mRNA levels in HeLa cells treated with different concentrations of STM2457. (C) Western blot analysis of gE and p-IRF3 in HeLa cells treated with various concentrations of PD98059. (D) qPCR analysis of PRV-*gE* mRNA levels in HeLa cells treated with different concentrations of PD98059. (E) Western blot was conducted to analyze the protein levels of gE and p-IRF3 in HeLa cells treated with 12 μmol/mL STM2457 and 20 μmol/mL PD98059 for 24 h, followed by PRV infection for 18 h. (F) qPCR analysis of PRV-*gE* mRNA levels and **(G)** viral DNA copy number in HeLa cells treated with STM2457 and PD98059. The final results are presented as the normalized viral DNA copy number, with error bars representing the standard error of the mean. Data were shown as mean ± SD based on three independent experiments. * p < 0.05, *** p < 0.001, **** p < 0.0001 determined by two-tailed Student’s *t*-test. EV: Empty vector control, which contains no target sequence. V: Samples infected with PRV.

To further clarify the role of the ERK-METTL3 axis in antiviral innate immunity, we performed experiments using the MEK1/2 inhibitor PD98059 and the METTL3 inhibitor STM2457 [25,26]. First, we evaluated the cytotoxicity of various concentrations and exposure times of these inhibitors on HeLa cells. Finding no significant differences in cell viability, we selected three concentrations for each drug for subsequent experiments: STM2457 at 4, 8, and 12 µM, and PD98059 at 5, 10, and 20 µM (S9A and S9B Fig). After treating cells with these concentrations for 24 h, we infected them with PRV and collected samples for Western blot and qPCR analysis. We observed a concentration-dependent decrease in m^6^A modification levels (S10 Fig), confirming the effectiveness of the selected concentrations. Subsequently, we analyzed the protein and mRNA levels of PRV-*gE*, finding that *gE* expression increased with higher drug concentrations (Fig 8A and 8B). Similarly, PD98059 treatment led to a concentration-dependent increase in PRV-gE protein expression, with a significant elevation at 20 µM (Fig 8C and 8D). Additionally, we detected a reduction in p-IRF3 protein levels with both inhibitors, which possibly may facilitate viral replication (Fig 8A and 8C). To further investigate, we combined STM2457 (12 µM) and PD98059 (20 µM). This combination resulted in a decrease in p-IRF3 protein levels and a substantial increase in both protein and mRNA levels of gE compared to the control group (Fig 8E and 8F). The combination treatment also significantly increased the viral DNA copy number (Fig 8G). Therefore, these findings suggest that inhibiting ERK2 and METTL3 significantly enhances PRV replication *in vitro*.

### The inhibitors of ERK2 and METTL3 significantly promote PRV replication *in vivo*

**Fig 9:**
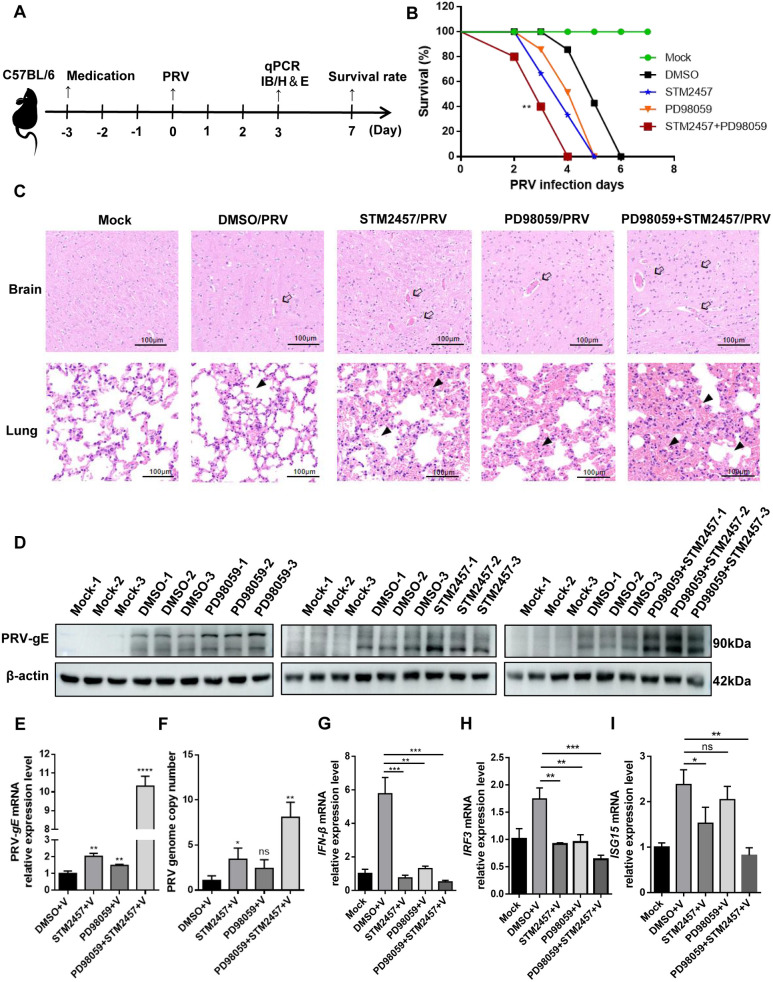
STM2457 and PD98059 enhanced PRV replication *in vivo.* (A) Schematic representation of the experimental approach for inhibitor administration and PRV infection in mice. (B) Mice were intraperitoneally injected with DMSO, STM2457 (25 mg/kg), or PD98059 (20 mg/kg) three days prior to PRV infection. Mice were then infected intraperitoneally with PRV (10^5^ TCID_50_ per mouse). Survival rates were monitored daily for 7 days post-infection (dpi) (n = 5 per group). (C) Hematoxylin and eosin (H&E) staining of brain and lung sections from mice. Arrows indicate increased fibrous components in the brain parenchyma, accompanied by hemorrhagic phenomena and severe inflammatory reactions. Triangles indicate significant increases in pulmonary consolidation, characterized by lung dysfunction and granulocyte infiltration. Scale bars, 100 μm. (D) Mice were treated as in B. On day 3, western blot analysis of PRV-gE protein levels in the brain (n = 3). (E) Mice were treated as in (B). On day 3, qPCR analysis of PRV-*gE* mRNA levels in the brain. (F) Mice were treated as in B. On day 3, qPCR analysis of viral DNA copy number in the brain. (G) Mice were treated as in B. On day 3, qPCR analysis of *IFN-β* mRNA levels in the brain. (H) Mice were treated as in B. On day 3, qPCR analysis of *IRF3* mRNA levels in the brain. (I) Mice were treated as in B. On day 3, qPCR analysis of I*SG15* mRNA levels in the brain. The final results are presented as the normalized viral DNA copy number, with error bars representing the standard error of the mean. Data were shown as mean ± SD based on three independent experiments. * p < 0.05, ** p < 0.01, *** p < 0.001, **** p < 0.0001 determined by two-tailed Student’s *t*-test. V: Samples infected wi*t*h PRV. The mock group was non-PRV infection and non-transfection group.

**Fig 10:**
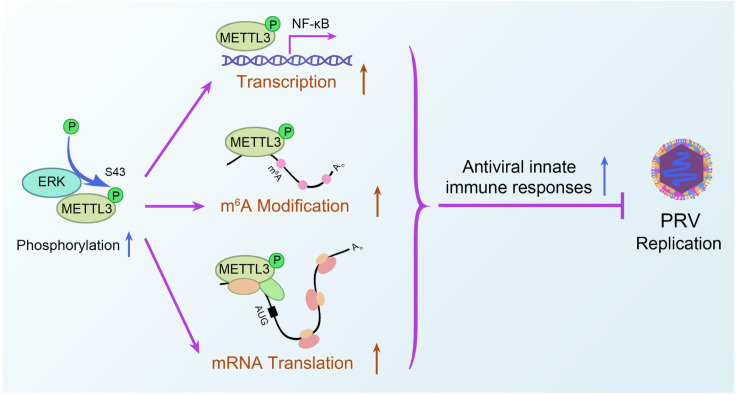
(A) Hypothetical model showing how the ERK-METTL3 axis suppresses α-herpesvirus proliferation through the antiviral innate immune pathway.

To evaluate the physiological role of the ERK-METTL3 axis in antiviral immunity, we conducted an *in vivo* study using C57BL/6 mice (Fig 9A). Mice were administered either PD98059, an MEK1/2 inhibitor (10 mg/kg), or STM2457, a METTL3 inhibitor (25 mg/kg) [27,28]. We set up five groups: Mock, DMSO, STM2457, PD98059, and a combination of STM2457 and PD98059. Treatments were given via intraperitoneal injections using solubilizers, with PRV infection commencing after three days of continuous use. Following infection, status and survival rates were monitored daily to generate survival curves. Our results showed that the combination treatment group experienced notably more severe damage than the control. Notably, mice in this group began dying on day two post-infection, while deaths in the control group started on day four. This indicates that inhibiting ERK2 and METTL3 significantly enhanced viral replication and accelerated mortality in mice (Fig 9B). Histological analysis of lung and brain tissues was performed three days post-infection using H&E staining. In brain tissues, the single drug treatment groups exhibited stronger inflammatory responses and cerebral vascular congestion compared to the DMSO-treated control. The combination group displayed the most severe lesions, including neuronal damage with nuclear condensation, basophilic granule deposition, and extensive lymphocyte infiltration. In lung tissues, the single drug treatment groups showed more severe parenchymal lesions, vascular dilation with hemorrhage, and inflammatory cell infiltration than the DMSO group. The combination group had the most severe lesions, marked by extensive infiltration of alveolar macrophages and monocytes, a near absence of alveolar epithelial cells, and almost complete lung function loss (Fig 9C). We further analyzed PRV-gE protein and mRNA levels, along with viral DNA copy number in brain tissues. As anticipated, levels of gE protein and mRNA were significantly higher in the combination treatment group than in the DMSO control group (Fig 9D and 9E), as were viral DNA copy number (Fig 9F). Previous *in vitro* studies showed that the ERK-METTL3 axis regulates IFN-β, affecting viral replication. Here, we examined whether this mechanism influences viral replication through interferon signaling pathways *in vivo*. Interestingly, *IFN-β* (Fig 9G), *IRF3* (Fig 9H), and *ISG15* (Fig 9I) levels were significantly lower in the combination group, suggesting that ERK2 and METTL3 inhibitors promote viral replication *in vivo* by modulating IFN expression, leading to organ damage and faster mortality in mice. Overall, ERK2 phosphorylation of METTL3 not only activates NF-κB to regulate IFN production but also increases m^6^A modification and enhances translation. This cascade amplification effect triggers an antiviral innate immune response that inhibits PRV replication (Fig 10).

## Discussion

Post-translational modifications (PTMs) involve the covalent addition of specific chemical groups to amino acid side chains [29], occurring either enzymatically or non-enzymatically. Over 600 types of PTMs have been identified to date, including phosphorylation, SUMOylation, ubiquitination, and acetylation [30]. Phosphorylation is particularly important in viral infections, where it supports viral processes like replication and proliferation [31]. Recent studies show that ERK phosphorylates METTL3 at the S43, S50, and S525 sites, reducing its ubiquitination by interacting with USP5. This interaction stabilizes METTL3 and increases m^6^A levels on mRNA [4]. Additionally, TBK1, a key kinase in antiviral pathways, phosphorylates METTL3 at the S67 site, which enhances protein translation. This modification increases m^6^A on IRF3 mRNA, stabilizing it and regulating IFN expression [13]. Our study confirmed that ERK-mediated phosphorylation of METTL3 plays a significant role in controlling antiviral innate immune responses (Fig 5A). Interestingly, recent findings suggest that the PRV-encoded US3 protein phosphorylates METTL3, significantly reducing m^6^A modification levels without notably affecting viral replication [24]. We propose that phosphorylation of METTL3 may be a strategic mechanism used by host or viral kinases in virus-host interactions. Our findings demonstrate that ERK2-mediated phosphorylation of METTL3 at S43 is essential for its regulatory effect on viral replication. S43 is located at the N-terminus of METTL3, where structural analysis indicates that this region, including its vicinity, closely interacts with a specific domain of METTL14, forming a key binding interface [32]. Mutation studies have shown that alterations in residues within this region, such as S43, destabilize the complex and indirectly reduce enzyme activity. Thus, phosphorylation at S43 may directly affect either catalytic activity or complex stability. In contrast, the C-terminal site S525 may be involved in RNA binding or other functions unrelated to interferon signaling. We hypothesize that modifications at S50 and S525 may depend on specific subcellular localization or ERK isoform activity, with this study primarily focusing on ERK2. Although phosphorylation at S50 and S525 did not exhibit significant functionality in this experimental framework, their roles in distinct physiological or pathological contexts warrant further investigation. Although METTL3 also has phosphorylation sites like S2, S348, and S350, these appear largely dispensable for its basic functions [33]. The existence of additional regulatory phosphorylation sites on METTL3 during viral infection remains an open question. Beyond phosphorylation, SUMOylation, ubiquitination, and acetylation also regulate the innate immune IFN signaling pathway [8,31]. We suggest that further exploration of the connections between METTL3 PTMs and their role in innate immunity could offer valuable insights for future research.

In this study, it is noteworthy that although PRV activates ERK2 and ERK2-mediated phosphorylation of METTL3 enhances m^6^A modification, overall m^6^A levels decrease during PRV infection in line with earlier research [24] (Fig 7A). Previous studies have shown that PRV broadly represses cellular transcription, preventing the expression of host genes, including inducible genes essential for antiviral responses [34]. PRV encodes over 70 proteins, several of which suppress host gene expression. Meanwhile, UL41 facilitates IRF3 degradation by recruiting the selective autophagy receptor Tollip [35], thereby suppressing IFN signaling. Additionally, EP0 reduces cellular IRF9 levels by inhibiting IRF9 mRNA, impairing IFN-induced gene transcription [36]. In this study, PRV infection decreased METTL3 expression, as evidenced by both in *vitro* and *in* *vivo* experiments (Fig 1). We hypothesize that a PRV-encoded protein may similarly regulate METTL3 degradation. Further research is essential to elucidate the specific molecular mechanisms underlying this regulation. Additionally, PRV activates the NF-κB pathway via the DNA damage response but suppresses NF-κB-dependent gene expression, limiting its antiviral effects [37]. NF-κB, a key transcription factor, plays a central role in several cellular signaling pathways, particularly in response to viral infections [22]. Our findings indicate that PRV activates the NF-κB pathway to regulate IFN expression, thereby influencing viral replication (Fig 6A and 6B). This interaction illustrates a dynamic balance between virus and host, where the virus seeks to manipulate host processes to enhance its replication, while the host responds with robust immune defenses to inhibit infection. Regarding cellular senescence, the role of METTL3 and its interaction with NF-κB signaling has been partly elucidated. Specifically, under certain conditions, METTL3 can relocalize to the nucleus and bind directly to the *NF-*κ*B* gene promoter. This binding intensifies NF-κB activation, initiating a cascade of downstream signaling, particularly in genes associated with immune responses [20]. Although we did not specifically examine how ERK2 phosphorylates METTL3 to activate NF-κB, we analyzed the cytokine profile stemming from this interaction. Our results showed that ERK2-mediated phosphorylation of METTL3 significantly upregulated mRNA levels of *IL-1β*, *IL-8*, *IL-6* and *TNF-α* (Fig 6C–6F). Moreover, other research has shown that Budding uninhibited by benzimidazole 1 (BUB1) interacts with METTL3 to induce m^6^A modification of mRNA, further enhancing NF-κB activation [38]. This finding provides new insights for our research into the ERK2-mediated phosphorylation of METTL3 in NF-κB activation. Furthermore, the interaction between ERK2 and METTL3 in antiviral immunity suggests that these molecules may independently yet synergistically influence viral replication. Activation of the ERK signaling pathway enhances IFN-β production during porcine coronavirus infection, thereby inhibiting viral replication [39]. Simultaneously, METTL3 stabilizes IRF3 mRNA via m^6^A modification, a process facilitated by TBK1-mediated activation. Additionally, METTL3 regulates IFN-β production through multiple innate immune pathways, including TLR3, TLR4, and TLR7/8 [13]. Collectively, in this study, when functioning as an integrated pathway, the ERK-METTL3 axis concurrently enhances IFN production, suppresses PRV replication, and strengthens antiviral defense through coordinated dual mechanisms.

Epitranscriptomic modifications such as m^6^A, significantly influence the regulation of the type I IFN response, which is crucial for both activation and inhibition [9]. The m^6^A modification plays a pivotal role in regulating mRNA stability and translation, thereby influencing gene expression [40]. Viral proteins can manipulate host m^6^A modifications by altering the expression and activity of m^6^A-related proteins, which in turn affects cytokine levels, suppresses immune cell proliferation and activity, and reduces stress responses, ensuring efficient viral replication. Studies have revealed that m^6^A modification dysregulation is linked to the innate immune response during viral infections [11]. For instance, m^6^A modification sites exist on the mRNA of Influenza A Virus (IAV), promoting viral replication by influencing the stability and translation of viral mRNA [41]. Additionally, the PRV-encoded US3 protein causes the dissociation of METTL3 from chromatin, leading to a decrease in m^6^A modification levels [24]. Our research confirmed this trend, with decreased m^6^A levels observed following PRV infection (Fig 7A). This decrease may serve as an escape mechanism evolved by the virus. Moreover, m^6^A plays multiple antiviral roles during infection period [42]. One important function of m^6^A involves enhancing the antiviral response, which depends on the targeted transcripts and the downstream effects of m^6^A on these RNA molecules. For instance, activation of METTL3 by TBK1 mediates m^6^A modification, stabilizing IRF3 expression and enhancing the innate immune response [13]. METTL3 and METTL14 can upregulate IFITM1 expression by adding m^6^A modifications to the 3’UTR of specific ISGs (IFN-stimulated genes), and the m^6^A methyltransferase complex can enhance the antiviral activity of type I IFNs [9]. In our study, the ERK-METTL3 axis increased m^6^A modification levels and inhibited PRV replication by regulating IFN, indicating an immune response mounted by the host against viral invasion. This is a complex battle between the virus and the host, with viruses potentially enhancing their replication through regulating m^6^A modifications, while hosts generate innate immune responses through their own m^6^A modifications to combat viral invasion.

Immunoprecipitation combined with mass spectrometry assays identified METTL3 interacting proteins, which are involved in protein translation. TBK1 promotes protein translation by phosphorylating METTL3 at the S67 site, enhancing its interaction with translation-related proteins [13]. Our study found that ERK2 phosphorylation of METTL3 significantly enhances protein translation. Notably, mutating the S43 phosphorylation site of METTL3 results in a significant reduction in protein translation compared to wild-type METTL3 (Fig 7G), suggesting that ERK2-mediated phosphorylation at S43 plays a crucial role in regulating protein translation. METTL3 methylates the transcript of elongation factor 1α (eEF1A), participating in the regulation of mRNA translation [43]. Additionally, METTL3 was also found to interact with eIF3C, a component of the eukaryotic translation initiation factor [44]. Therefore, we hypothesize that ERK2 phosphorylation of METTL3 may enhance protein translation through its regulation of eIF3C. Although we found ERK2 phosphorylation of METTL3 at the S43 site to regulate protein translation, we cannot rule out the possibility that ERK2 may also phosphorylate METTL3 at the S67 site. The different phosphorylation sites may alter METTL3 structure to enhance interaction with translation-related proteins [45]. The ability of m^6^A modification to regulate many pathological and physiological processes by controlling the expression of mRNAs and non-coding RNAs (ncRNAs) is of particular interest. METTL3, among other methyltransferases, catalyzes the methylation of three main types of ncRNAs: circular RNAs (circRNAs), long non-coding RNAs (lncRNAs), and microRNAs (miRNAs) [46]. Among these, circRNAs are ncRNAs that exist in a circular structure and typically lack the potential to translate into proteins or peptides. However, after m^6^A modification, they can enhance translation. For example, m^6^A-methylated circMAP3K4, catalyzed by METTL3, can be translated into circMAP3K4-455aa with the help of IGF2 BP1 [47]. lncRNAs are at least 200 nucleotides in length, and serve as intermediaries in genetic information transfer and perform various regulatory functions. METTL3 catalyzes the methylation of lncRNAs, increasing their expression levels by reducing RNA degradation and stabilizing RNA transcripts [48]. miRNAs are small ncRNAs that regulate gene expression by promoting mRNA cleavage or translation inhibition [49]. METTL14 and FTO-mediated m^6^A modification regulate PCV2 replication by influencing the maturation of miRNAs [50]. Therefore, we speculate that ERK2-mediated phosphorylation of METTL3 may promote cellular protein translation through interacting with translation-related proteins or regulating m^6^A modifications on mRNAs and ncRNAs.

As a core m^6^A methyltransferase, METTL3 plays a multifaceted role in viral infection, including the regulation of viral replication and participation in innate immune responses [51,52]. It is known that both ERK2 and METTL3 are involved in viral replication [53,54]. Given that overexpression of ERK2 and METTL3 impedes PRV replication, while their knockdown facilitates it (Fig 2D–2I), we explored the potential impact of ERK and METTL3 inhibitors on PRV replication. We selected PD98059, a specific inhibitor of ERK2, to block its phosphorylation and activation by inhibiting MEK (MAPK/ERK kinase) activity [55]. Additionally, we used STM2457, an effective and highly specific inhibitor of METTL3 catalytic activity [26]. We administered these drugs in combination to validate the effect of the ERK-METTL3 axis on PRV replication in mice. The PD98059 dose was 10 mg/kg, which has been reported as non-toxic in mice when administered intraperitoneally [27]. For STM2457, we used a dose of 25 mg/kg, which did not cause significant changes in body weight compared to the control group in mice [28]. These drug concentrations were deemed safe for our experiments. Our study revealed significantly higher morbidity and mortality in the combination treatment group, with the most severe lesions observed in infected brain and lung tissues (Fig 9C). This clearly indicates that PD98059 and STM2457 significantly enhanced viral replication in mice, thereby accelerating pathogenesis. Since a METTL3 activator is unavailable, we have not evaluated small molecule agonists to validate the antiviral effect of the ERK-METTL3 axis [56]. It is important to note that PRV is a herpesvirus primarily infecting pigs. However, our current validation of drug effects has been limited to mouse models. To ensure the clinical applicability of our findings, future trials using pigs as the primary experimental animals for validation will provide more accurate insights into the efficacy and safety of the drugs in the natural host of PRV. A limitation of this study is the reliance on overexpression models in some experiments. Overexpression can overload the protein expression system, induce cellular stress, and introduce biases. However, knockout cell line models were also used to validate the findings, and the results remained consistent.

In summary, our study showed that the ERK-METTL3 axis activates NF-κB to regulate IFN expression, increases m^6^A modification and protein translation levels, and amplifies the IFN signaling pathway, thereby affecting PRV replication. The phosphorylation of METTL3 at the S43 site by ERK2 is crucial for regulating antiviral innate immune responses. This research provides the first evidence of the ERK-METTL3 axis’s role in innate immunity and clarifies its mechanism, enhancing our understanding of herpesvirus biology and offering a potential therapeutic target for PRV prevention and control.

## Materials and methods

### Ethics statement

All animal experiments were conducted in accordance with protocols approved by the ethics and animal welfare committee of Henan Agricultural University, following the National Guide for the Care and Use of Laboratory Animals (approval number: SYXK-YU-2021–0003).

### Cells

Human cervical cancer cells (HeLa), human embryonic kidney cells (HEK-293T), swine testicle cells (ST), and porcine kidney epithelial cells (PK-15) were maintained in our laboratory. These cells were cultured in Dulbecco’s Modified Eagle’s Medium (DMEM, Solarbio, Beijing, China) supplemented with 10% Fetal Bovine Serum (FBS, Gibco, USA) at 37°C in a 5% CO_2_ cell incubator.

### Plasmids and antibodies

The wild-type (WT) ERK2 and METTL3 genes were amplified from HEK-293T mRNA (cDNA) and cloned into 3 × FLag-cmv-14-ERK2 and pCAGGS-HA vectors, respectively. shERK2 and shMETTL3 were cloned by primer annealing into pGPU6-GFP-Neo. ERK2 mutants were generated by directed mutation based on WT ERK2. METTL3 mutants and truncations were generated by directed mutation or truncation based on WT METTL3. The 3 × FLag-cmv-14-ERK2 and pCAGGS-HA vectors were preserved in our laboratory. The NF-κB-Luciferase Reporter Plasmid and Thymidine Kinase Plasmid were generously provided by Professor Yu Linyang from the College of Veterinary Medicine, Henan Agricultural University.

The antibodies used and their sources are listed below:

- Rabbit Anti-ERK1/2, Rabbit Anti-PERK1/2, Rabbit Anti-MEK, Rabbit Anti-P-MEK, Rabbit Anti-IRF3, Rabbit Anti-N^6^-methyladenosine, HRP Goat Anti-Mouse IgG (H + L) were purchased from ABclonal Biotechnology Co., Ltd. (Wuhan, China).
- Rabbit Anti-METTL3 was purchased from Abmart Shanghai Co., Ltd. (Shanghai, China).
- Rabbit Anti-HA, Mouse Anti-DDDDK-tag, Rabbit Anti-β-actin, and HRP Goat Anti-Rabbit IgG (H + L) were purchased from Proteintech Group, Inc. (Wuhan, China).
- gE antibody was kindly provided by the Key Laboratory of Animal Immunology (Zhengzhou, China).

### Chemicals

PD98059, the ERK2 inhibitor, and STM2457, the METTL3 inhibitor, were both dissolved in DMSO and purchased from InvivoChem (Guangzhou, China). Puromycin dihydrochloride (MCE, NJ, USA) was dissolved in ultra-pure water and used at a concentration of 10 μg/mL to evaluate protein translation.

### Viruses

Pseudorabies virus (PRV), strain HeNLH (GenBank: MT775883.1), was provided by the Key Laboratory of Animal Immunology, Henan Academy of Agricultural Sciences. PRV was propagated in PK-15 cells, and the supernatants were stored as a stock solution. The virus titer was determined by titration to achieve 10^5^ TCID_50_ in PK-15 cells.

### Mice and *in vivo* virus infection and drug treatment

In this study, eight-week-old female C57BL/6 mice were used. All animal procedures adhered to the Guide for the Use of Laboratory Animals of the Chinese Association for Laboratory Animal Science. The mice were obtained from Zhengzhou Huiji District Huaxing Laboratory Animal Farm and maintained under pathogen-free conditions. Mice were infected with PRV at a dose of 10^5^ TCID_50_ via intraperitoneal injection. They were sacrificed at 3 or 5 dpi, and their brains and spleens were harvested. Negative control mice were also included. PD98059 (20 mg/kg) and STM2457 (25 mg/kg) were administered intraperitoneally. The drugs were dissolved in a vehicle containing 10% DMSO, 40% PEG300, 5% Tween 80, and 45% saline. After drug administration, mouse survival was monitored, and mortality was recorded (n = 5). Additionally, organs were harvested at 3 dpi for histological section preparation and examination (n = 3).

### Western blot

The 2 × SDS loading buffer was used to lyse the cells. The lysates were then boiled at 98°C for 15 min. The protein was separated using 7.5%, 10%, and 12.5% SDS-polyacrylamide gels. Electrophoresis was conducted at 80 V for 20 min, followed by 150 V for 40 min. Subsequently, the proteins were transferred to polyvinylidene difluoride (PVDF) membranes (Millipore, Massachusetts, USA) using a transfer apparatus at 250 mA for 90 min. The membranes were blocked with 5% skim milk, washed with TBST (TBS containing 0.1% Tween-20), and incubated overnight at 4°C with the primary antibody. After three washes with TBST, the membranes were incubated with HRP-conjugated Goat Anti-Rabbit IgG (H + L) antibody for 1 h at room temperature. Following four washes with TBST, the membranes were developed using the Amersham Imager 680 (General Electric, New York, USA).

### CRISPR-Cas9 mediated knockout

The CRISPR-Cas9 system was employed to knockout the endogenous *ERK2* and *METTL3* genes in HEK-293T cells. ERK2-sgRNA and METTL3-sgRNA plasmids were successfully constructed and co-transfected with pMD2.G and psPAX2 into HEK-293T cells to generate a lentiviral library. At 36 h post-transfection, cells exhibited morphological changes, including rounding and slowed growth. To enrich successfully transfected cells, selection was performed using 2 mg/mL puromycin for one week. The efficiency of gene knockout was assessed by Western blot, confirming the reduction or absence of the targeted endogenous proteins. Both polyclonal populations with reduced protein expression and monoclonal cell lines with complete gene knockout were identified and isolated.

### Dual-luciferase reporter assays

HeLa cells seeded on a 48-well plate were transfected with 125 ng of the luciferase reporter plasmid and various expression plasmids or an empty vector. As an internal control, 25 ng of pRL-TK was transfected simultaneously. Twelve hours post-transfection, cells were infected with PRV (MOI = 0.4), with a negative control established. Reporter gene activity was analyzed using the Dual Luciferase Reporter 1000 Analysis System (Promega Biotech Co., Ltd, USA).

### Quantitative real-time PCR

Total RNA was extracted from cultured cells and tissue using the RNA-Easy Isolation Reagent (R701, Vazyme Biotech Co., Ltd., China). The RNA concentration was measured with a Thermo NanoDrop2000/2000C spectrophotometer (Thermo Fisher Scientific Co., Ltd., USA). cDNA was reverse-transcribed from 500 ng of total RNA using the HiScript II 1st Strand cDNA Synthesis Kit (R212-02, Vazyme, China). For qRT-PCR, 1/20 volume of the cDNA was used as the template with the ChamQ Universal SYBR qPCR Master Mix (Q311-02, Vazyme, China). The qRT-PCR reactions were performed on a VeritiPro PCR instrument (A48141, Thermo Fisher Scientific Co., Ltd., USA). The mRNA levels of target genes were quantified using the 2^-ΔΔCT^ method, with primer sequences listed in S1 Table.

### PRV genome copy number quantification

Genomic DNA was isolated from PRV-infected HeLa cells for analysis using qPCR to detect both PRV viral DNA and cellular DNA. Following quantification, a standard curve was created by plotting the cycle threshold (Ct) values against the known copy numbers. Specifically, Pseudorabies virus *gB* and glyceraldehyde -phosphate dehydrogenase (*GAPDH*) were cloned into the pMD19-T plasmid vector. Standard curves were generated by plotting Ct values against copy numbers after qPCR. The copy numbers of gB and GAPDH in the samples were measured using the primers gB-F and gB-R, or GAPDH-F and GAPDH-R, respectively, and calculated using the aforementioned standard curves. This standard curve facilitated the calculation of the copy numbers for both viral and cellular DNA. To determine the standardized viral DNA copy number, the viral DNA copy number was divided by the cellular DNA copy number. Subsequently, the standardized viral DNA copy number from the experimental groups was compared to that of the control group by division, ensuring accurate normalization. The final results, presented as the normalized viral DNA copy number, include error bars that represent the standard error of the mean.

### Confocal microscopy

At 24 h post-transfection, HeLa cells were fixed with 4% paraformaldehyde for 30 min and permeabilized with 0.1% Triton X-100 for 20 min. Next, the cells were then blocked with 1% BSA for 1 h. After blocking, the cells were washed three times with 1 × PBS. Mouse monoclonal anti-FLAG and rabbit monoclonal anti-HA antibodies (1:1000 dilution in 10% FBS) were added to each well, and the cells were incubated for 1 h at room temperature. Following this, the cells were washed three times with 1 × PBS. Then, secondary antibodies, 488 Goat anti-mouse (1:500) and 594 Goat anti-rabbit (1:500) diluted in 10% FBS, and 300 μL of the diluted antibody was added to each well, and incubated for 1 h at room temperature. Finally, 200 μL of DAPI (1:2000 dilution in PBS) was added to stain nuclei for 2–5 min.

### Co-immunoprecipitation assay

HEK-293T cells were cultured to 70% confluence and co-transfected with a mixture of plasmids (10 μg total), including 3 × Flag-CMV-14 empty vector, pCAGGS-HA-METTL3, and 3 × Flag-CMV-14-ERK2. PEI was used at a DNA to PEI ratio of 1:2. After 24 h, cells were lysed in NP40 buffer with protease inhibitors. Protein interactions were evaluated via co-immunoprecipitation using magnetic beads conjugated to Flag or HA tags, followed by Western blot after SDS-PAGE denaturation.

### m^6^A dot blots

Total RNA from HeLa cells was denatured at 72°C for 5 min and then cooled rapidly on ice. Next, 2 mg of RNA was applied to a Hybond-N+ membrane (GE Health, NC, USA). The membrane was UV crosslinked for 5 min in SCIENTZ03-II (SCIENTZ, Ningbo, China), stained with 0.02% methylene blue, and scanned using a Molecular Imaging Gel Doc XR System (Bio-Rad, CA, USA) to visualize total RNA content. After washing with 1% SDS in 0.2 × SSC buffer, membranes were blocked with 5% skimmed milk and incubated overnight at 4°C with a specific m^6^A antibody (Abclonal, Wuhan, China). Membranes were then washed thrice with PBST, incubated with HRP-conjugated secondary antibody for 1 h, washed again thrice with PBST, and imaged using an Amersham Imager 680 (General Electric, New York, USA).

### SUnSET assay

Cells were incubated with 10 μg/mL puromycin for 60 min, washed with ice-cold PBS, and lysed using RIPA lysis buffer. Equal amounts of cell lysates were analyzed by Western blot using an anti-puromycin antibody to detect protein synthesis. Signals were normalized using GAPDH as a loading control.

### CCK-8 assay

HeLa cells in the logarithmic growth phase were seeded into a 96-well culture plate and allowed to adhere for 24h. After adding the drug, 10 μl of CCK-8 detection solution was added to each well at 0, 6, 12, 24, and 36 h. The cells were then incubated for 1 h in a cell incubator. The absorbance was measured at a wavelength of 450 nm, and the data was subsequently analyzed.

### Histopathology

Mouse brain and lung tissues were fixed overnight in universal neutral tissue fixative, trimmed, and embedded in paraffin. Paraffin blocks were sectioned at a 3–4 μm thickness using a microtome. The sections were flattened on a 40 °C water bath and transferred onto slides. The slides were dried in a 60°C oven and stained with hematoxylin-eosin (H&E). Prepared histological sections underwent whole-slide scanning and were examined using image analysis software (Caseviewer 2.4, Hungary).

### Statistical analysis

All experiments were repeated more than three times. Data are presented as the mean ± standard deviation (SD). The data were analyzed using GraphPad Prism 9.0 (GraphPad, CA, USA). An unpaired two-tailed Students’ t-test was used for comparing two groups, with statistical significance set at p < 0.05. For mouse survival studies, Kaplan-Meier survival curves were generated and analyzed using the log-rank test.

## Supporting information

S1 Fig Analysis of PRV infection in mouse spleen samples. (A) Samples were processed at 0, 12, 18, and 24 h after PRV (MOI = 0.4) infection, and the localization changes of ERK2 in cells were verified using laser confocal microscopy. (B) Western blot was used to analyze protein expression changes in the MAPK signaling pathway, METTL3, and PRV-gE in mouse spleen samples before PRV infection and at 3 dpi and 5 dpi. Samples were collected from the spleens of C57BL/6 mice.
(TIF)

S2 Fig Impact of METTL3 and ERK2 on *gB* mRNA Levels. (A) The endogenous ERK2 distribution was detected between the Mock and PRV 18 h after infection (MOI = 0.4). (B) HeLa cells were transfected with empty vector, 3 × FLag-CMV-14-ERK2, pCAGGS-HA-METTL3, and 3 × FLag-CMV-14-ERK2 + pCAGGS-HA-METTL3, respectively. After 12 h, cells were infected with PRV (MOI = 0.4), and a blank control group was established. Cell samples were collected 24 h post-infection, and PRV-*gB* mRNA expression was detected by qPCR. (C) HeLa cells were transfected with empty vectors, sh-ERK2, sh-METTL3, and sh-ERK2 + sh-METTL3, respectively. After 12 h, cells were infected with PRV (MOI = 0.4), and a blank control group was established. Cell samples were collected 24 h post-infection, and PRV-*gB* mRNA expression was detected by qPCR. Data were shown as mean ± SD based on three independent experiments. ** p < 0.01, *** p < 0.001, **** p < 0.0001 determined by two-tailed Student’s *t*-test. EV: Empty vector control, which contains no target sequence. sh: shRNA empty vector control, which contains the shRNA scaffold but no specific target sequence.
(TIF)

S3 Fig Verification of sgRNA Knockout Efficiency in HeLa cells. (A) METTL3-sgRNA, (B)ERK2-sgRNA, and the empty lentilCRISPR-v2 vector were co-transfected with two packaging helper plasmids, pSPAX2 and pMD2.G, into HEK-293T cells. After lentivirus production, the viruses were used to infect HeLa cells. qPCR was then used to detect the knockdown efficiency of METTL3 and ERK2. Similar to (A), cells were treated and analyzed using Western blot to assess the knockdown efficiency of METTL3 (C) and ERK2 (D). Data are presented as mean ± SD from three independent experiments. ** p < 0.01, *** p < 0.001, **** p < 0.0001, determined by two-tailed Student’s *t*-test.
(TIF)

S4 Fig The impact of sgRNA-mediated knockout cell lines on viral replication during PRV replication. (A) ERK2-sgRNA, METTL3-sgRNA, and the empty lentilCRISPR-v2 vector were co-transfected with two packaging helper plasmids, pSPAX2 and pMD2.G, into HEK-293T cells. After lentivirus production, the viruses were used to infect HeLa cells. In the sg-ERK2 and sg-METTL3 cell lines, PRV (MOI = 0.4) infection was conducted. Cell samples were collected 24 h later, and PRV-*gE* mRNA expression was detected by qPCR. (B) Similar to (A), PRV (MOI = 0.4) infection was conducted in the sg-ERK2 and sg-METTL3 cell lines. Cell samples were collected 24 h later, and PRV-*gB* mRNA expression was detected by qPCR. (C) Similar to (A), PRV (MOI = 0.4) infection was conducted in the sg-ERK2 and sg-METTL3 cell lines. Cell samples were collected 24 h later, and PRV-gE protein expression was detected by Western blot. **(D)** Same as **(A)** treated cells, in the sg-ERK2 and sg-METTL3 cell lines, PRV (MOI = 0.4) infection was conducted in the sg-ERK2 and sg-METTL3 cell lines. Cell samples were collected 24 h later, and viral DNA copy number was detected by qPCR. lentiCRISPR-v2: Lentiviral CRISPR-Cas9 System Version 2. The lentiCRISPR-v2 vector without any sgRNA sequence inserted was used as a control. The final results are presented as the normalized viral DNA copy number, with error bars representing the standard error of the mean.Data were shown as mean ± SD based on three independent experiments. * p < 0.05, ** p < 0.01, *** p < 0.001, **** p < 0.0001 determined by two-tailed Student’s *t*-test.
(TIF)

S5 Fig Verification of sgRNA Knockout Efficiency in PK-15 Cells (A) ERK2-sgRNA, METTL3-sgRNA, and the empty lentiCRISPR-v2 vector were co-transfected with two packaging helper plasmids, pSPAX2 and pMD2.G, into HEK-293T cells. METTL3-sgRNA, ERK2-sgRNA, and the empty lentiCRISPR-v2 vector were co-transfected with two packaging helper plasmids, pSPAX2 and pMD2.G, into HEK-293T cells. Following lentivirus production, PK-15 cells were infected with the viruses. Knockdown efficiency of METTL3 **(A)** and ERK2 **(B)** was assessed by qPCR. Western blot was used to evaluate METTL3 **(C)** and ERK2 **(D)** knockdown efficiency. Data are presented as mean ± SD from three independent experiments. *p < 0.05, **p < 0.01, ***p < 0.001 determined by two-tailed Student’s *t*-test.
(TIF)

S6 Fig Effect of sgRNA-mediated knockout on PRV replication in PK-15 cells. (A) ERK2-sgRNA, METTL3-sgRNA, and the empty lentiCRISPR-v2 vector were co-transfected with two packaging helper plasmids, pSPAX2 and pMD2.G into HEK-293T cells. After lentivirus production, PK-15 cells were infected. PRV infection (MOI = 0.4) was performed in sg-ERK2 and sg-METTL3 cell lines, and PRV-*gE* mRNA levels were quantified by qPCR 24 h post-infection. (B) Similar to (A), PRV infection (MOI = 0.4) was conducted in sg-ERK2 and sg-METTL3 cell lines, and PRV-*gB* mRNA levels were quantified by qPCR 24 h post-infection. (C) Similar to (A), PRV infection (MOI = 0.4) was performed in sg-ERK2 and sg-METTL3 cell lines. Cell samples were collected 24 h later, and PRV-gE protein expression was assessed by Western blot 24 h post-infection. lentiCRISPR-v2: Lentiviral CRISPR-Cas9 System Version 2. The lentiCRISPR-v2 vector without an inserted sgRNA sequence served as a control. (D) Similar to (A), PRV infection (MOI = 0.4) was performed in sg-ERK2 and sg-METTL3 cell lines. Cell samples were collected 24 h later, viral DNA copy number were determined by qPCR 24 h post-infection. lentiCRISPR-v2: Lentiviral CRISPR-Cas9 System Version 2. The lentiCRISPR-v2 vector without an inserted sgRNA sequence served as a control. The final results are expressed as normalized viral DNA copy number, with error bars representing the standard error of the mean. Data are presented as mean ± SD from three independent experiments. *p < 0.05, **p < 0.01, ***p < 0.001 determined by two-tailed Student’s *t*-test.
(TIF)

S7 Fig The effects of METTL3 phosphorylation site mutations on PRV replication. qPCR analysis of the effect of co-transfecting ERK2 with WT METTL3 and its mutants S43, S50, and S525 on PRV-*gB*. Data are shown as mean ± SD based on three independent experiments. ** p < 0.01, *** p < 0.001 determined by two-tailed Student’s *t*-test. EV: Empty vector control, which contains no target sequence.
(TIF)

S8 Fig The effects of ERK mutants on PRV replication. qPCR analysis of the impact of co-transfecting METTL3 with ERK2 and its mutants, ERK2-321N, on PRV-*gB*. Data are presented as mean ± SD from three independent experiments. Statistical significance was determined using a two-tailed Student’s *t*-test: ** p < 0.01, *** p < 0.001. EV: Empty vector control, which contains no target sequence.
(TIF)

S9 Fig Validation of the efficacy of STM2457 and PD98059 in HeLa cells. (A) HeLa cells were treated with varying concentrations of STM2457 (0–12 μmol/mL) for 0–36 h. Cell viability was assessed using the CCK-8 assay. (B) HeLa cells were treated with varying concentrations of PD98059 (0–20 μM) for 0–36 h. Cell viability was assessed using the CCK-8 assay.
(TIF)

S10 Fig STM2457 inhibited m^6^A levels in a concentration-dependent manner. m^6^A dot blot analysis was conducted to evaluate m^6^A levels in HeLa cells treated with STM2457 (0–12 μM).
(TIF)

S1 Table Primers used in this study. (DOCX)

## References

[1] Connolly SA, Jardetzky TS, Longnecker R. The structural basis of herpesvirus entry. Nat Rev Microbiol. 2021;19(2):110–21. doi: 10.1038/s41579-020-00448-w 33087881 PMC8579738

[2] Tsai K, Cullen BR. Epigenetic and epitranscriptomic regulation of viral replication. Nat Rev Microbiol. 2020;18(10):559–70. doi: 10.1038/s41579-020-0382-3 32533130 PMC7291935

[3] Shi H, Wei J, He C. Where, When, and How: Context-Dependent Functions of RNA Methylation Writers, Readers, and Erasers. Mol Cell. 2019;74(4):640–50. doi: 10.1016/j.molcel.2019.04.025 31100245 PMC6527355

[4] Sun H-L, Zhu AC, Gao Y, Terajima H, Fei Q, Liu S, et al. Stabilization of ERK-Phosphorylated METTL3 by USP5 Increases m6A Methylation. Mol Cell. 2020;80(4):633–647.e7. doi: 10.1016/j.molcel.2020.10.026 33217317 PMC7720844

[5] Liu J, Yue Y, Han D, Wang X, Fu Y, Zhang L, et al. A METTL3-METTL14 complex mediates mammalian nuclear RNA N6-adenosine methylation. Nat Chem Biol. 2014;10(2):93–5. doi: 10.1038/nchembio.1432 24316715 PMC3911877

[6] Jia G, Fu Y, Zhao X, Dai Q, Zheng G, Yang Y, et al. N6-methyladenosine in nuclear RNA is a major substrate of the obesity-associated FTO. Nat Chem Biol. 2011;7(12):885–7. doi: 10.1038/nchembio.687 22002720 PMC3218240

[7] Zheng G, Dahl JA, Niu Y, Fedorcsak P, Huang C-M, Li CJ, et al. ALKBH5 is a mammalian RNA demethylase that impacts RNA metabolism and mouse fertility. Mol Cell. 2013;49(1):18–29. doi: 10.1016/j.molcel.2012.10.015 23177736 PMC3646334

[8] Rehwinkel J, Gack MU. RIG-I-like receptors: their regulation and roles in RNA sensing. Nat Rev Immunol. 2020;20(9):537–51. doi: 10.1038/s41577-020-0288-3 32203325 PMC7094958

[9] McFadden MJ, McIntyre ABR, Mourelatos H, Abell NS, Gokhale NS, Ipas H, et al. Post-transcriptional regulation of antiviral gene expression by N6-methyladenosine. Cell Rep. 2021;34(9):108798. doi: 10.1016/j.celrep.2021.108798 33657363 PMC7981787

[10] Rubio RM, Depledge DP, Bianco C, Thompson L, Mohr I. RNA m6 A modification enzymes shape innate responses to DNA by regulating interferon β. Genes Dev. 2018;32(23–24):1472–84. doi: 10.1101/gad.319475.118 30463905 PMC6295168

[11] Winkler R, Gillis E, Lasman L, Safra M, Geula S, Soyris C, et al. m6A modification controls the innate immune response to infection by targeting type I interferons. Nat Immunol. 2019;20(2):173–82. doi: 10.1038/s41590-018-0275-z 30559377

[12] Qiu W, Zhang Q, Zhang R, Lu Y, Wang X, Tian H, et al. N6-methyladenosine RNA modification suppresses antiviral innate sensing pathways via reshaping double-stranded RNA. Nat Commun. 2021;12(1):1582. doi: 10.1038/s41467-021-21904-y 33707441 PMC7952553

[13] Chen J, Wei X, Wang X, Liu T, Zhao Y, Chen L, et al. TBK1-METTL3 axis facilitates antiviral immunity. Cell Rep. 2022;38(7):110373. doi: 10.1016/j.celrep.2022.110373 35172162

[14] Lavoie H, Gagnon J, Therrien M. ERK signalling: a master regulator of cell behaviour, life and fate. Nat Rev Mol Cell Biol. 2020;21(10):607–32. doi: 10.1038/s41580-020-0255-7 32576977

[15] DuShane JK, Maginnis MS. Human DNA Virus Exploitation of the MAPK-ERK Cascade. Int J Mol Sci. 2019;20(14):3427. doi: 10.3390/ijms20143427 31336840 PMC6679023

[16] Liu Q, Wang X, Xie C, Ding S, Yang H, Guo S, et al. A Novel Human Acute Encephalitis Caused by Pseudorabies Virus Variant Strain. Clin Infect Dis. 2021;73(11):e3690–700. doi: 10.1093/cid/ciaa987 32667972

[17] Pomeranz LE, Reynolds AE, Hengartner CJ. Molecular biology of pseudorabies virus: impact on neurovirology and veterinary medicine. Microbiol Mol Biol Rev. 2005;69(3):462–500. doi: 10.1128/MMBR.69.3.462-500.2005 16148307 PMC1197806

[18] Wang P, Doxtader KA, Nam Y. Structural Basis for Cooperative Function of Mettl3 and Mettl14 Methyltransferases. Mol Cell. 2016;63(2):306–17. doi: 10.1016/j.molcel.2016.05.041 27373337 PMC4958592

[19] Crow YJ, Stetson DB. The type I interferonopathies: 10 years on. Nat Rev Immunol. 2022;22(8):471–83. doi: 10.1038/s41577-021-00633-9 34671122 PMC8527296

[20] Liu P, Li F, Lin J, Fukumoto T, Nacarelli T, Hao X, et al. m6A-independent genome-wide METTL3 and METTL14 redistribution drives the senescence-associated secretory phenotype. Nat Cell Biol. 2021;23(4):355–65. doi: 10.1038/s41556-021-00656-3 33795874 PMC8035315

[21] Seth RB, Sun L, Ea C-K, Chen ZJ. Identification and characterization of MAVS, a mitochondrial antiviral signaling protein that activates NF-kappaB and IRF 3. Cell. 2005;122(5):669–82. doi: 10.1016/j.cell.2005.08.012 16125763

[22] Baker RG, Hayden MS, Ghosh S. NF-κB, inflammation, and metabolic disease. Cell Metab. 2011;13(1):11–22. doi: 10.1016/j.cmet.2010.12.008 21195345 PMC3040418

[23] Liu Y, You Y, Lu Z, Yang J, Li P, Liu L, et al. N6-methyladenosine RNA modification-mediated cellular metabolism rewiring inhibits viral replication. Science. 2019;365(6458):1171–6. doi: 10.1126/science.aax4468 31439758

[24] Jansens RJJ, Verhamme R, Mirza AH, Olarerin-George A, Van Waesberghe C, Jaffrey SR, et al. Alphaherpesvirus US3 protein-mediated inhibition of the m6A mRNA methyltransferase complex. Cell Rep. 2022;40(3):111107. doi: 10.1016/j.celrep.2022.111107 35858564 PMC9347262

[25] Wang Z-Y, Li Y-Q, Guo Z-W, Zhou X-H, Lu M-D, Xue T-C, et al. ERK1/2-HNF4α axis is involved in epigallocatechin-3-gallate inhibition of HBV replication. Acta Pharmacol Sin. 2020;41(2):278–85. doi: 10.1038/s41401-019-0302-0 31554961 PMC7468327

[26] Yankova E, Blackaby W, Albertella M, Rak J, De Braekeleer E, Tsagkogeorga G, et al. Small-molecule inhibition of METTL3 as a strategy against myeloid leukaemia. Nature. 2021;593(7860):597–601. doi: 10.1038/s41586-021-03536-w 33902106 PMC7613134

[27] Di Paola R, Galuppo M, Mazzon E, Paterniti I, Bramanti P, Cuzzocrea S. PD98059, a specific MAP kinase inhibitor, attenuates multiple organ dysfunction syndrome/failure (MODS) induced by zymosan in mice. Pharmacol Res. 2010;61(2):175–87. doi: 10.1016/j.phrs.2009.09.008 19819333

[28] Zhang R, Chen P, Wang Y, Zeng Z, Yang H, Li M, et al. Targeting METTL3 enhances the chemosensitivity of non-small cell lung cancer cells by decreasing ABCC2 expression in an m6A-YTHDF1-dependent manner. Int J Biol Sci. 2024;20(12):4750–66. doi: 10.7150/ijbs.97425 39309428 PMC11414383

[29] Jensen ON. Interpreting the protein language using proteomics. Nat Rev Mol Cell Biol. 2006;7(6):391–403. doi: 10.1038/nrm1939 16723975

[30] Huang X, Feng Z, Liu D, Gou Y, Chen M, Tang D, et al. PTMD 2.0: an updated database of disease-associated post-translational modifications. Nucleic Acids Res. 2025;53(D1):D554–63. doi: 10.1093/nar/gkae850 39329270 PMC11701619

[31] Chan YK, Gack MU. Viral evasion of intracellular DNA and RNA sensing. Nat Rev Microbiol. 2016;14(6):360–73. doi: 10.1038/nrmicro.2016.45 27174148 PMC5072394

[32] Wang X, Feng J, Xue Y, Guan Z, Zhang D, Liu Z, et al. Structural basis of N(6)-adenosine methylation by the METTL3-METTL14 complex. Nature. 2016;534(7608):575–8. doi: 10.1038/nature18298 27281194

[33] Schöller E, Weichmann F, Treiber T, Ringle S, Treiber N, Flatley A, et al. Interactions, localization, and phosphorylation of the m6A generating METTL3-METTL14-WTAP complex. RNA. 2018;24(4):499–512. doi: 10.1261/rna.064063.117 29348140 PMC5855951

[34] Romero N, Wuerzberger-Davis SM, Van Waesberghe C, Jansens RJ, Tishchenko A, Verhamme R, et al. Pseudorabies Virus Infection Results in a Broad Inhibition of Host Gene Transcription. J Virol. 2022;96(13):e0071422. doi: 10.1128/jvi.00714-22 35730976 PMC9278110

[35] Yan Z, Yue J, Zhang Y, Hou Z, Li D, Yang Y, et al. Pseudorabies virus VHS protein abrogates interferon responses by blocking NF-κB and IRF3 nuclear translocation. Virol Sin. 2024;39(4):587–99. doi: 10.1016/j.virs.2024.05.009 38823782 PMC11401465

[36] Wang M, Liu Y, Qin C, Lang Y, Xu A, Yu C, et al. Pseudorabies Virus EP0 Antagonizes the Type I Interferon Response via Inhibiting IRF9 Transcription. J Virol. 2022;96(13):e0217121. doi: 10.1128/jvi.02171-21 35708311 PMC9278115

[37] Romero N, Favoreel HW. Pseudorabies Virus Infection Triggers NF-κB Activation via the DNA Damage Response but Actively Inhibits NF-κB-Dependent Gene Expression. J Virol. 2021;95(24):e0166621. doi: 10.1128/JVI.01666-21 34613805 PMC8610585

[38] Wang K, Shen K, Wang J, Yang K, Zhu J, Chen Y, et al. BUB1 potentiates gastric cancer proliferation and metastasis by activating TRAF6/NF-κB/FGF18 through m6A modification. Life Sci. 2024;353:122916. doi: 10.1016/j.lfs.2024.122916 39025206

[39] Li M, Zhang L, Zhou P, Zhang Z, Yu R, Zhang Y, et al. Porcine deltacoronavirus nucleocapsid protein interacts with the Grb2 through its proline-rich motifs to induce activation of the Raf-MEK-ERK signal pathway and promote virus replication. J Gen Virol. 2024;105(8):10.1099/jgv.0.002014. doi: 10.1099/jgv.0.002014 39136113 PMC12453405

[40] He PC, He C. m6 A RNA methylation: from mechanisms to therapeutic potential. EMBO J. 2021;40(3):e105977. doi: 10.15252/embj.2020105977 33470439 PMC7849164

[41] Courtney DG, Kennedy EM, Dumm RE, Bogerd HP, Tsai K, Heaton NS, et al. Epitranscriptomic Enhancement of Influenza A Virus Gene Expression and Replication. Cell Host Microbe. 2017;22(3):377-386.e5. doi: 10.1016/j.chom.2017.08.004 28910636 PMC5615858

[42] Imam H, Khan M, Gokhale NS, McIntyre ABR, Kim G-W, Jang JY, et al. N6-methyladenosine modification of hepatitis B virus RNA differentially regulates the viral life cycle. Proc Natl Acad Sci USA. 2018;115(35):8829–34. doi: 10.1073/pnas.1808319115 30104368 PMC6126736

[43] Jakobsson ME, Małecki JM, Halabelian L, Nilges BS, Pinto R, Kudithipudi S, et al. The dual methyltransferase METTL13 targets N terminus and Lys55 of eEF1A and modulates codon-specific translation rates. Nat Commun. 2018;9(1):3411. doi: 10.1038/s41467-018-05646-y 30143613 PMC6109062

[44] Masutani M, Sonenberg N, Yokoyama S, Imataka H. Reconstitution reveals the functional core of mammalian eIF3. EMBO J. 2007;26(14):3373–83. doi: 10.1038/sj.emboj.7601765 17581632 PMC1933396

[45] Colson BA, Thompson AR, Espinoza-Fonseca LM, Thomas DD. Site-directed spectroscopy of cardiac myosin-binding protein C reveals effects of phosphorylation on protein structural dynamics. Proc Natl Acad Sci USA. 2016;113(12):3233–8. doi: 10.1073/pnas.1521281113 26908877 PMC4812748

[46] Liu Z, Gao L, Cheng L, Lv G, Sun B, Wang G, et al. The roles of N6-methyladenosine and its target regulatory noncoding RNAs in tumors: classification, mechanisms, and potential therapeutic implications. Exp Mol Med. 2023;55(3):487–501. doi: 10.1038/s12276-023-00944-y 36854773 PMC10073155

[47] Duan J-L, Chen W, Xie J-J, Zhang M-L, Nie R-C, Liang H, et al. A novel peptide encoded by N6-methyladenosine modified circMAP3K4 prevents apoptosis in hepatocellular carcinoma. Mol Cancer. 2022;21(1):93. doi: 10.1186/s12943-022-01537-5 35366894 PMC8976336

[48] Yin H, Chen L, Piao S, Wang Y, Li Z, Lin Y, et al. M6A RNA methylation-mediated RMRP stability renders proliferation and progression of non-small cell lung cancer through regulating TGFBR1/SMAD2/SMAD3 pathway. Cell Death Differ. 2023;30(3):605–17. doi: 10.1038/s41418-021-00888-8 34628486 PMC9984538

[49] Cullen BR. MicroRNAs as mediators of viral evasion of the immune system. Nat Immunol. 2013;14(3):205–10. doi: 10.1038/ni.2537 23416678 PMC3642974

[50] Li C, Wu X, Yang Y, Shi J, Wang S, Li J, et al. METTL14 and FTO mediated m6A modification regulate PCV2 replication by affecting miR-30a-5p maturity. Virulence. 2023;14(1):2232910. doi: 10.1080/21505594.2023.2232910 37418592 PMC10332184

[51] Zhang X, Hao H, Ma L, Zhang Y, Hu X, Chen Z, et al. Methyltransferase-like 3 Modulates Severe Acute Respiratory Syndrome Coronavirus-2 RNA N6-Methyladenosine Modification and Replication. mBio. 2021;12(4):e0106721. doi: 10.1128/mBio.01067-21 34225491 PMC8437041

[52] Li N, Hui H, Bray B, Gonzalez GM, Zeller M, Anderson KG, et al. METTL3 regulates viral m6A RNA modification and host cell innate immune responses during SARS-CoV-2 infection. Cell Rep. 2021;35(6):109091. doi: 10.1016/j.celrep.2021.109091 33961823 PMC8090989

[53] Kim Y, Lee C. Extracellular signal-regulated kinase (ERK) activation is required for porcine epidemic diarrhea virus replication. Virology. 2015;484:181–93. doi: 10.1016/j.virol.2015.06.007 26115165 PMC7111633

[54] Burgess HM, Depledge DP, Thompson L, Srinivas KP, Grande RC, Vink EI, et al. Targeting the m6A RNA modification pathway blocks SARS-CoV-2 and HCoV-OC43 replication. Genes Dev. 2021;35(13–14):1005–19. doi: 10.1101/gad.348320.121 34168039 PMC8247602

[55] Brognard J, Dennis PA. Variable apoptotic response of NSCLC cells to inhibition of the MEK/ERK pathway by small molecules or dominant negative mutants. Cell Death Differ. 2002;9(9):893–904. doi: 10.1038/sj.cdd.4401054 12181740

[56] Selberg S, Blokhina D, Aatonen M, Koivisto P, Siltanen A, Mervaala E, et al. Discovery of Small Molecules that Activate RNA Methylation through Cooperative Binding to the METTL3-14-WTAP Complex Active Site. Cell Rep. 2019;26(13):3762–3771.e5. doi: 10.1016/j.celrep.2019.02.100 30917327

