# Supplementary figures and images for: ERK-METTL3 axis acts as a novel regulator of antiviral innate immunity combating pseudorabies virus infection
Source: PLoS Pathog. 2025 Aug 13;21(8):e1013234. doi: 10.1371/journal.ppat.1013234 (PMC12349697; doi:10.1371/journal.ppat.1013234)

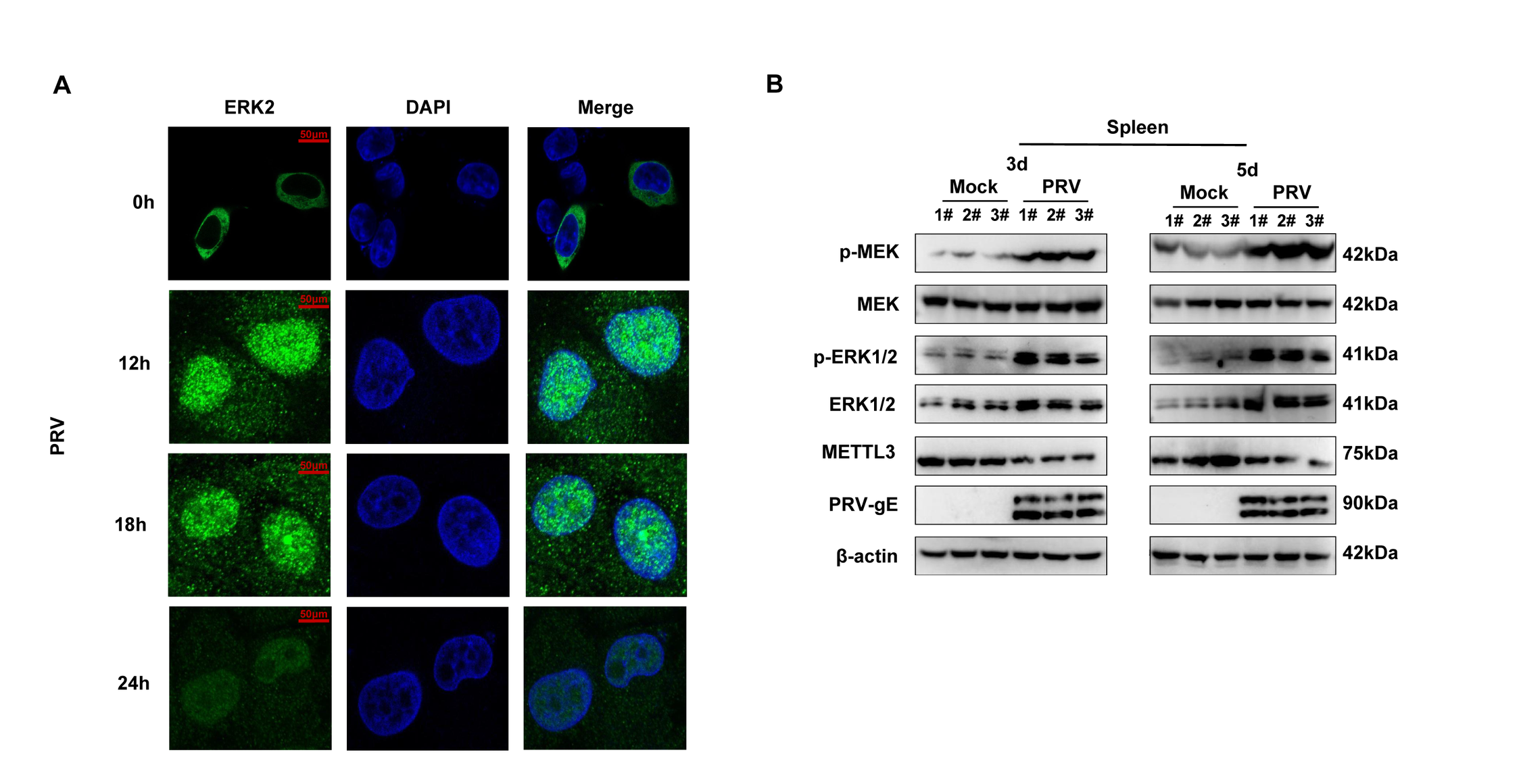

Supplement: S1 Fig — (A) Samples were processed at 0, 12, 18, and 24 h after PRV (MOI = 0.4) infection, and the localization changes of ERK2 in cells were verified using laser confocal microscopy. (B) Western blot was used to analyze protein expression changes in the MAPK signaling pathway, METTL3, and PRV-gE in mouse spleen samples before PRV infection and at 3 dpi and 5 dpi. Samples were collected from the spleens of C57BL/6 mice. (TIF) [file ppat.1013234.s001.tif]

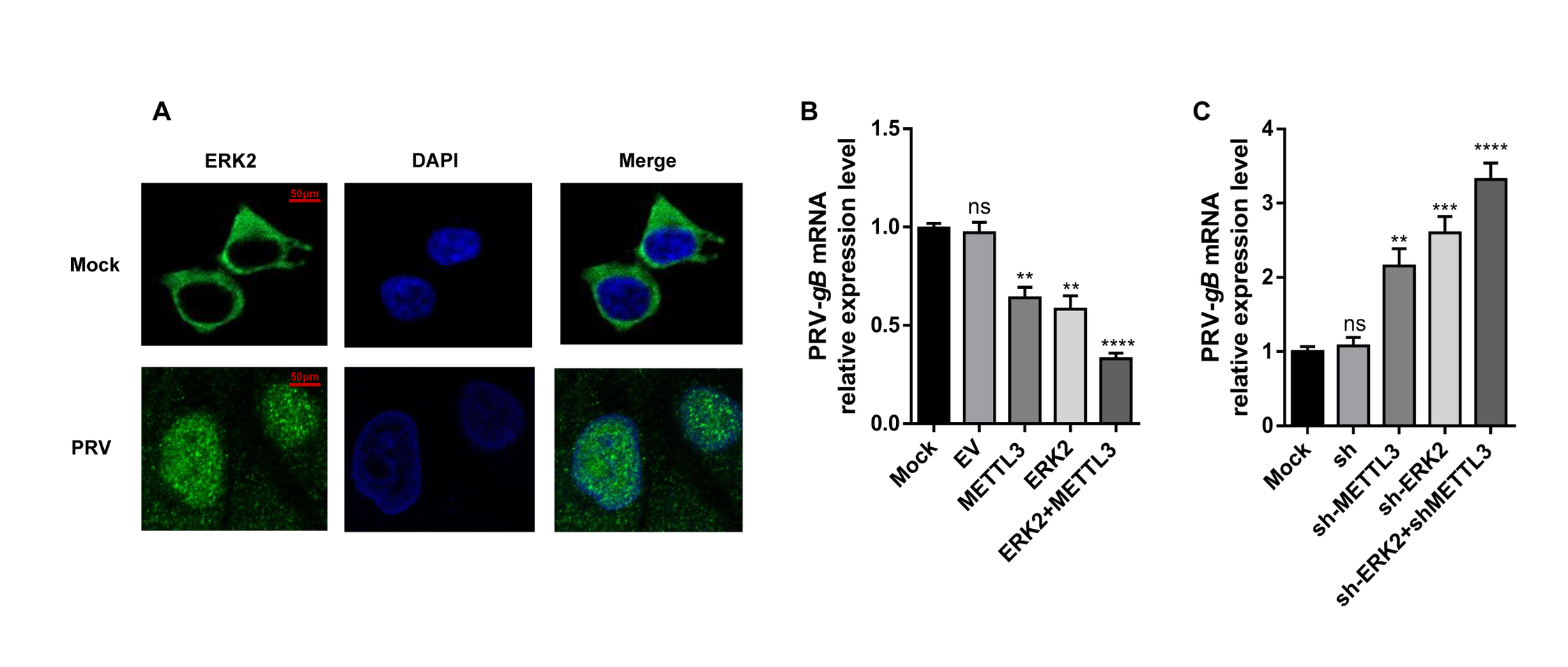

Supplement: S2 Fig — (A) The endogenous ERK2 distribution was detected between the Mock and PRV 18 h after infection (MOI = 0.4). (B) HeLa cells were transfected with empty vector, 3 × FLag-CMV-14-ERK2, pCAGGS-HA-METTL3, and 3 × FLag-CMV-14-ERK2 + pCAGGS-HA-METTL3, respectively. After 12 h, cells were infected with PRV (MOI = 0.4), and a blank control group was established. Cell samples were collected 24 h post-infection, and PRV-gB mRNA expression was detected by qPCR. (C) HeLa cells were transfected with empty vectors, sh-ERK2, sh-METTL3, and sh-ERK2 + sh-METTL3, respectively. After 12 h, cells were infected with PRV (MOI = 0.4), and a blank control group was established. Cell samples were collected 24 h post-infection, and PRV-gB mRNA expression was detected by qPCR. Data were shown as mean ± SD based on three independent experiments. ** p < 0.01, *** p < 0.001, **** p < 0.0001 determined by two-tailed Student’s t-test. EV: Empty vector control, which contains no target sequence. sh: shRNA empty vector control, which contains the shRNA scaffold but no specific target sequence. (TIF) [file ppat.1013234.s002.tif]

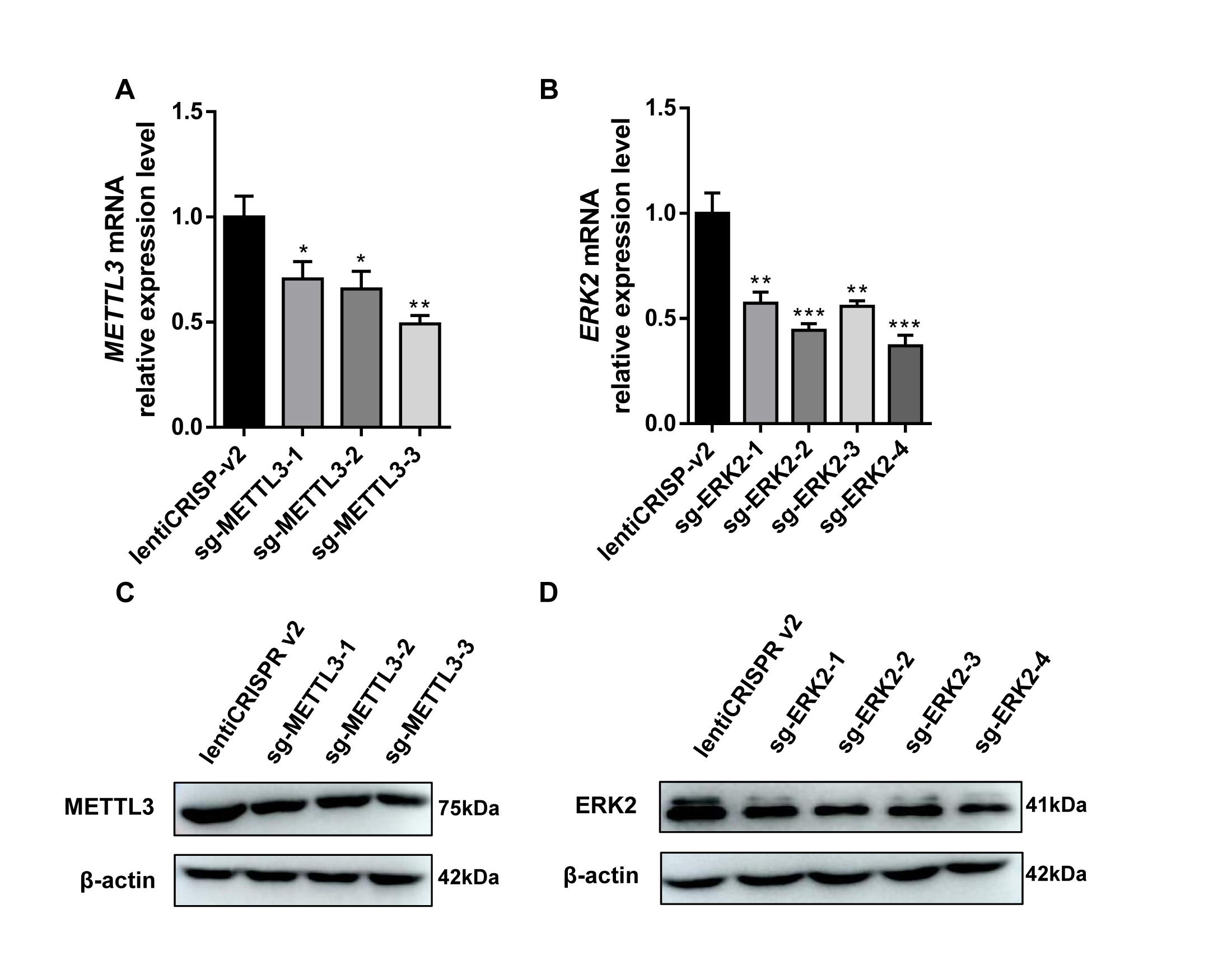

Supplement: S3 Fig — (A) METTL3-sgRNA, (B)ERK2-sgRNA, and the empty lentilCRISPR-v2 vector were co-transfected with two packaging helper plasmids, pSPAX2 and pMD2.G, into HEK-293T cells. After lentivirus production, the viruses were used to infect HeLa cells. qPCR was then used to detect the knockdown efficiency of METTL3 and ERK2. Similar to (A), cells were treated and analyzed using Western blot to assess the knockdown efficiency of METTL3 (C) and ERK2 (D). Data are presented as mean ± SD from three independent experiments. ** p < 0.01, *** p < 0.001, **** p < 0.0001, determined by two-tailed Student’s t-test. (TIF) [file ppat.1013234.s003.tif]

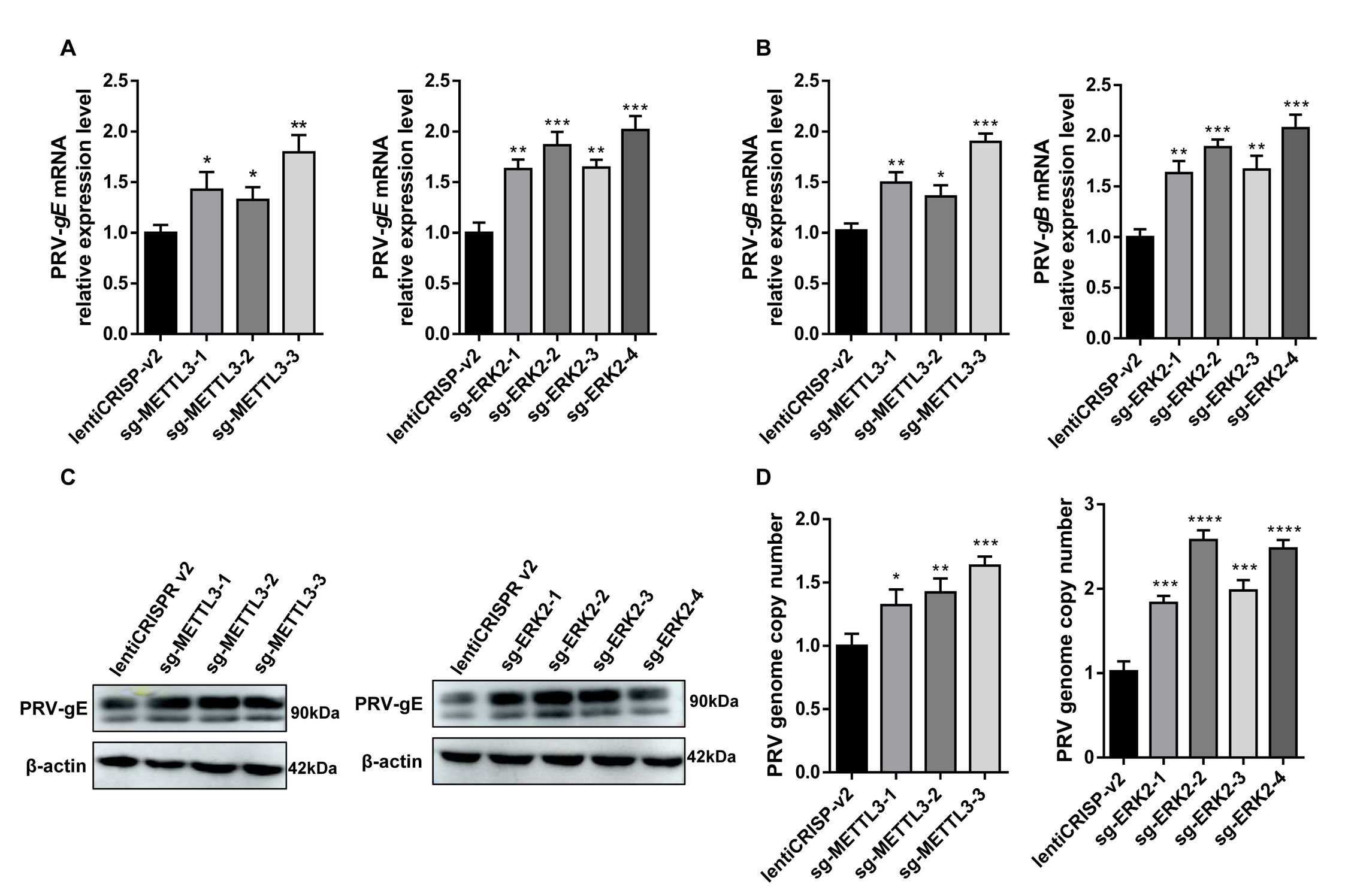

Supplement: S4 Fig — (A) ERK2-sgRNA, METTL3-sgRNA, and the empty lentilCRISPR-v2 vector were co-transfected with two packaging helper plasmids, pSPAX2 and pMD2.G, into HEK-293T cells. After lentivirus production, the viruses were used to infect HeLa cells. In the sg-ERK2 and sg-METTL3 cell lines, PRV (MOI = 0.4) infection was conducted. Cell samples were collected 24 h later, and PRV-gE mRNA expression was detected by qPCR. (B) Similar to (A), PRV (MOI = 0.4) infection was conducted in the sg-ERK2 and sg-METTL3 cell lines. Cell samples were collected 24 h later, and PRV-gB mRNA expression was detected by qPCR. (C) Similar to (A), PRV (MOI = 0.4) infection was conducted in the sg-ERK2 and sg-METTL3 cell lines. Cell samples were collected 24 h later, and PRV-gE protein expression was detected by Western blot. (D) Same as (A) treated cells, in the sg-ERK2 and sg-METTL3 cell lines, PRV (MOI = 0.4) infection was conducted in the sg-ERK2 and sg-METTL3 cell lines. Cell samples were collected 24 h later, and viral DNA copy number was detected by qPCR. lentiCRISPR-v2: Lentiviral CRISPR-Cas9 System Version 2. The lentiCRISPR-v2 vector without any sgRNA sequence inserted was used as a control. The final results are presented as the normalized viral DNA copy number, with error bars representing the standard error of the mean.Data were shown as mean ± SD based on three independent experiments. * p < 0.05, ** p < 0.01, *** p < 0.001, **** p < 0.0001 determined by two-tailed Student’s t-test. (TIF) [file ppat.1013234.s004.tif]

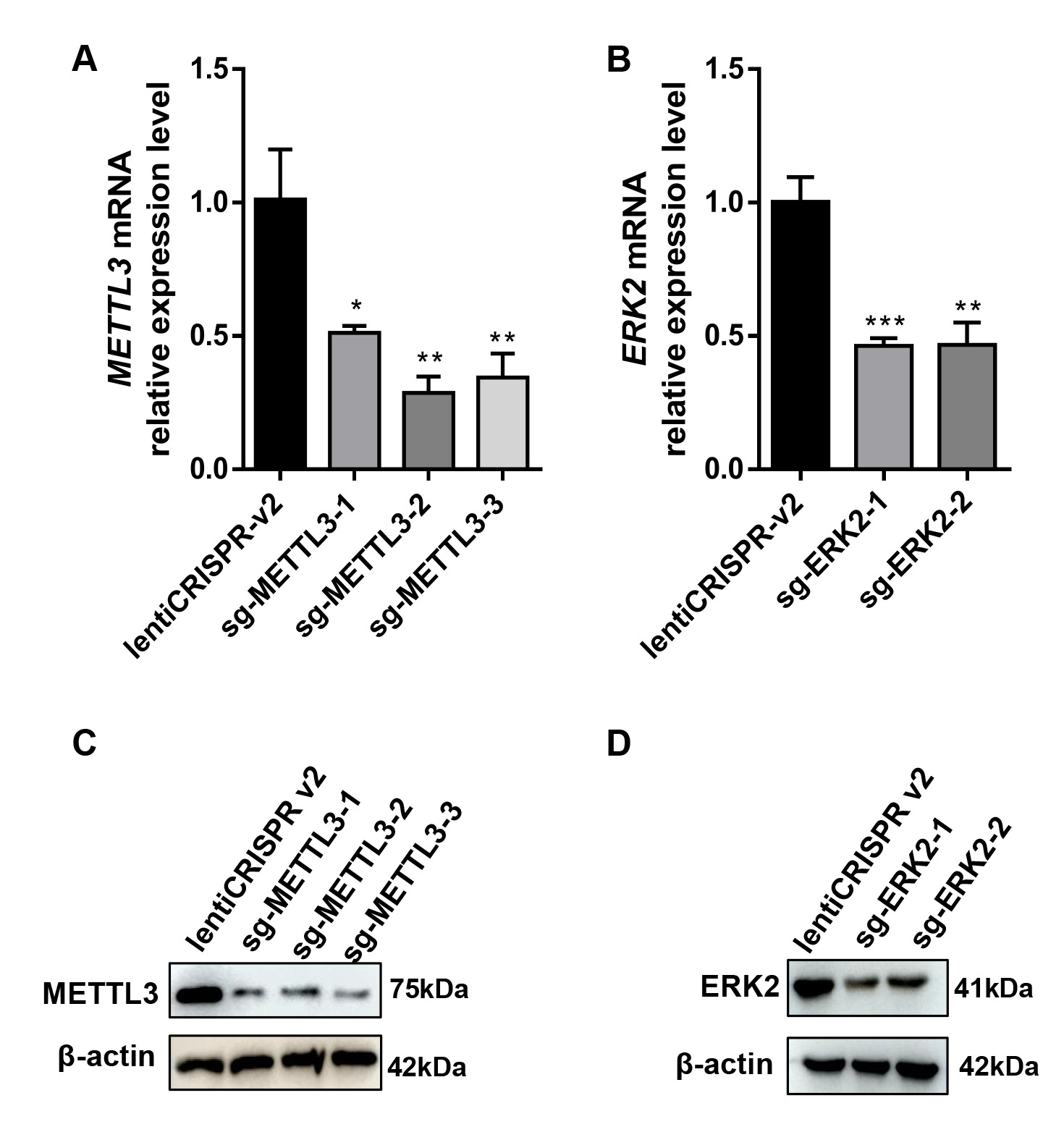

Supplement: S5 Fig — METTL3-sgRNA, ERK2-sgRNA, and the empty lentiCRISPR-v2 vector were co-transfected with two packaging helper plasmids, pSPAX2 and pMD2.G, into HEK-293T cells. Following lentivirus production, PK-15 cells were infected with the viruses. Knockdown efficiency of METTL3 (A) and ERK2 (B) was assessed by qPCR. Western blot was used to evaluate METTL3 (C) and ERK2 (D) knockdown efficiency. Data are presented as mean ± SD from three independent experiments. *p < 0.05, **p < 0.01, ***p < 0.001 determined by two-tailed Student’s t-test. (TIF) [file ppat.1013234.s005.tif]

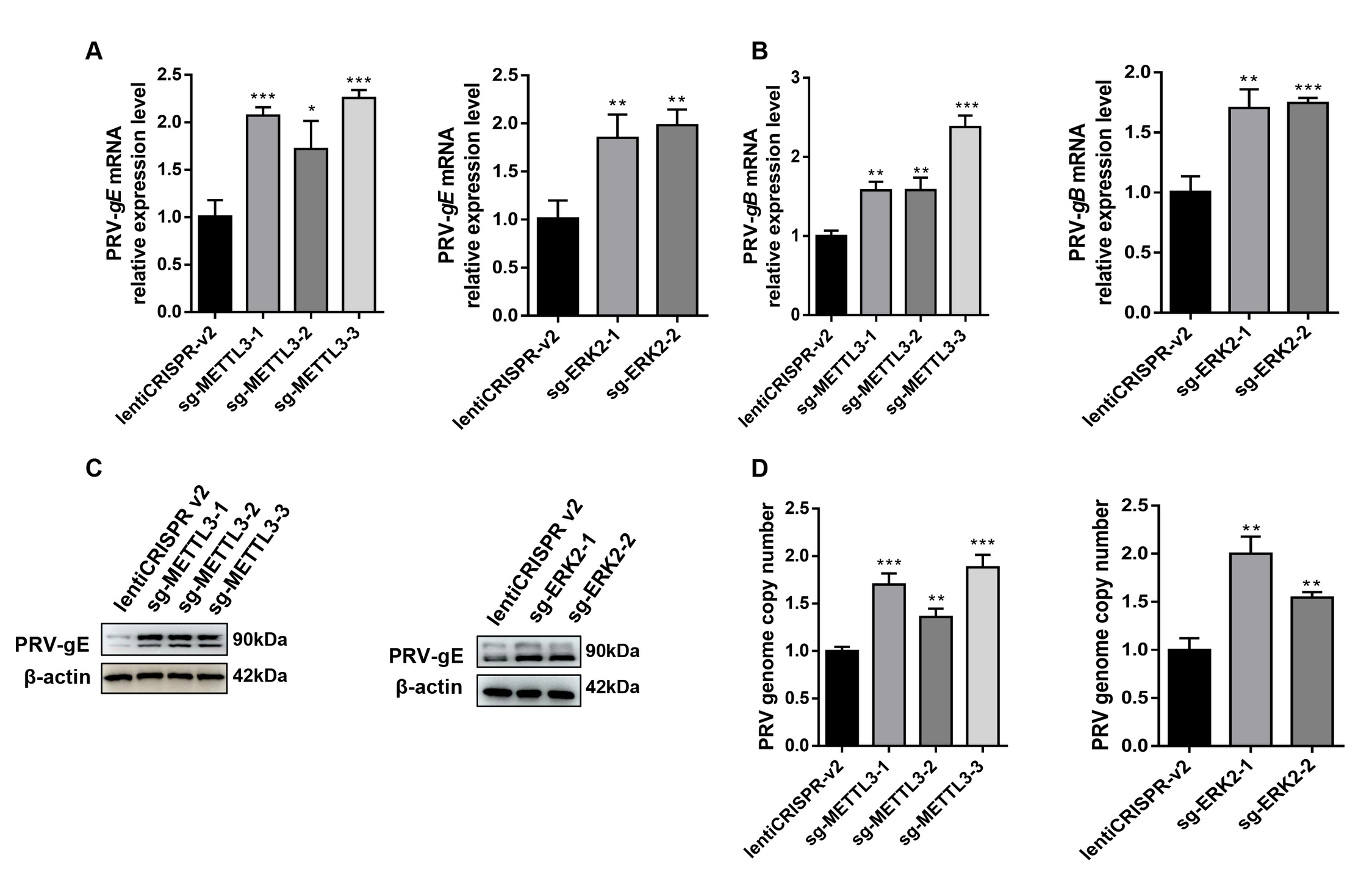

Supplement: S6 Fig — (A) ERK2-sgRNA, METTL3-sgRNA, and the empty lentiCRISPR-v2 vector were co-transfected with two packaging helper plasmids, pSPAX2 and pMD2.G into HEK-293T cells. After lentivirus production, PK-15 cells were infected. PRV infection (MOI = 0.4) was performed in sg-ERK2 and sg-METTL3 cell lines, and PRV-gE mRNA levels were quantified by qPCR 24 h post-infection. (B) Similar to (A), PRV infection (MOI = 0.4) was conducted in sg-ERK2 and sg-METTL3 cell lines, and PRV-gB mRNA levels were quantified by qPCR 24 h post-infection. (C) Similar to (A), PRV infection (MOI = 0.4) was performed in sg-ERK2 and sg-METTL3 cell lines. Cell samples were collected 24 h later, and PRV-gE protein expression was assessed by Western blot 24 h post-infection. lentiCRISPR-v2: Lentiviral CRISPR-Cas9 System Version 2. The lentiCRISPR-v2 vector without an inserted sgRNA sequence served as a control. (D) Similar to (A), PRV infection (MOI = 0.4) was performed in sg-ERK2 and sg-METTL3 cell lines. Cell samples were collected 24 h later, viral DNA copy number were determined by qPCR 24 h post-infection. lentiCRISPR-v2: Lentiviral CRISPR-Cas9 System Version 2. The lentiCRISPR-v2 vector without an inserted sgRNA sequence served as a control. The final results are expressed as normalized viral DNA copy number, with error bars representing the standard error of the mean. Data are presented as mean ± SD from three independent experiments. *p < 0.05, **p < 0.01, ***p < 0.001 determined by two-tailed Student’s t-test. (TIF) [file ppat.1013234.s006.tif]

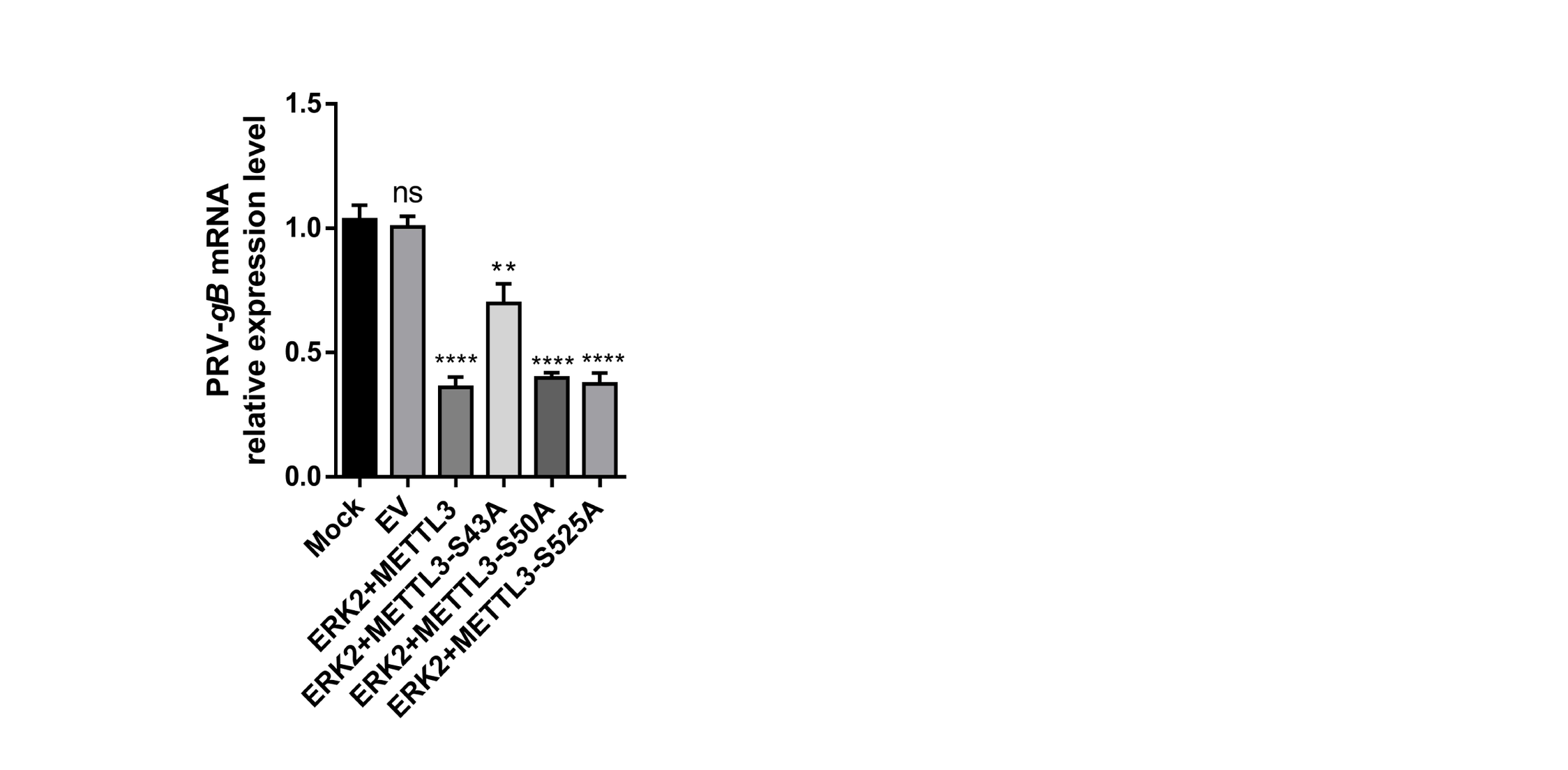

Supplement: S7 Fig — qPCR analysis of the effect of co-transfecting ERK2 with WT METTL3 and its mutants S43, S50, and S525 on PRV-gB. Data are shown as mean ± SD based on three independent experiments. ** p < 0.01, *** p < 0.001 determined by two-tailed Student’s t-test. EV: Empty vector control, which contains no target sequence. (TIF) [file ppat.1013234.s007.tif]

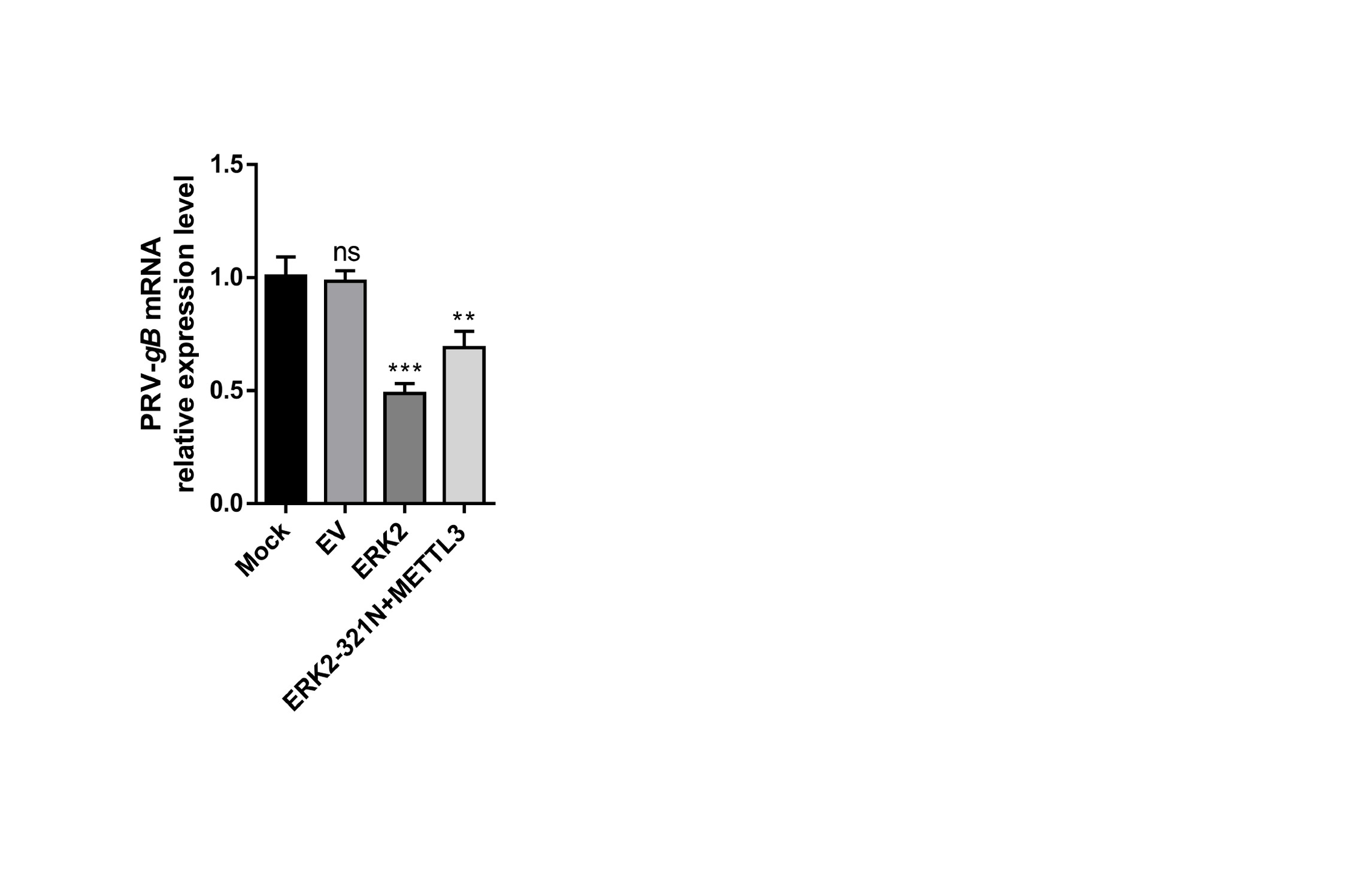

Supplement: S8 Fig — qPCR analysis of the impact of co-transfecting METTL3 with ERK2 and its mutants, ERK2-321N, on PRV-gB. Data are presented as mean ± SD from three independent experiments. Statistical significance was determined using a two-tailed Student’s t-test: ** p < 0.01, *** p < 0.001. EV: Empty vector control, which contains no target sequence. (TIF) [file ppat.1013234.s008.tif]

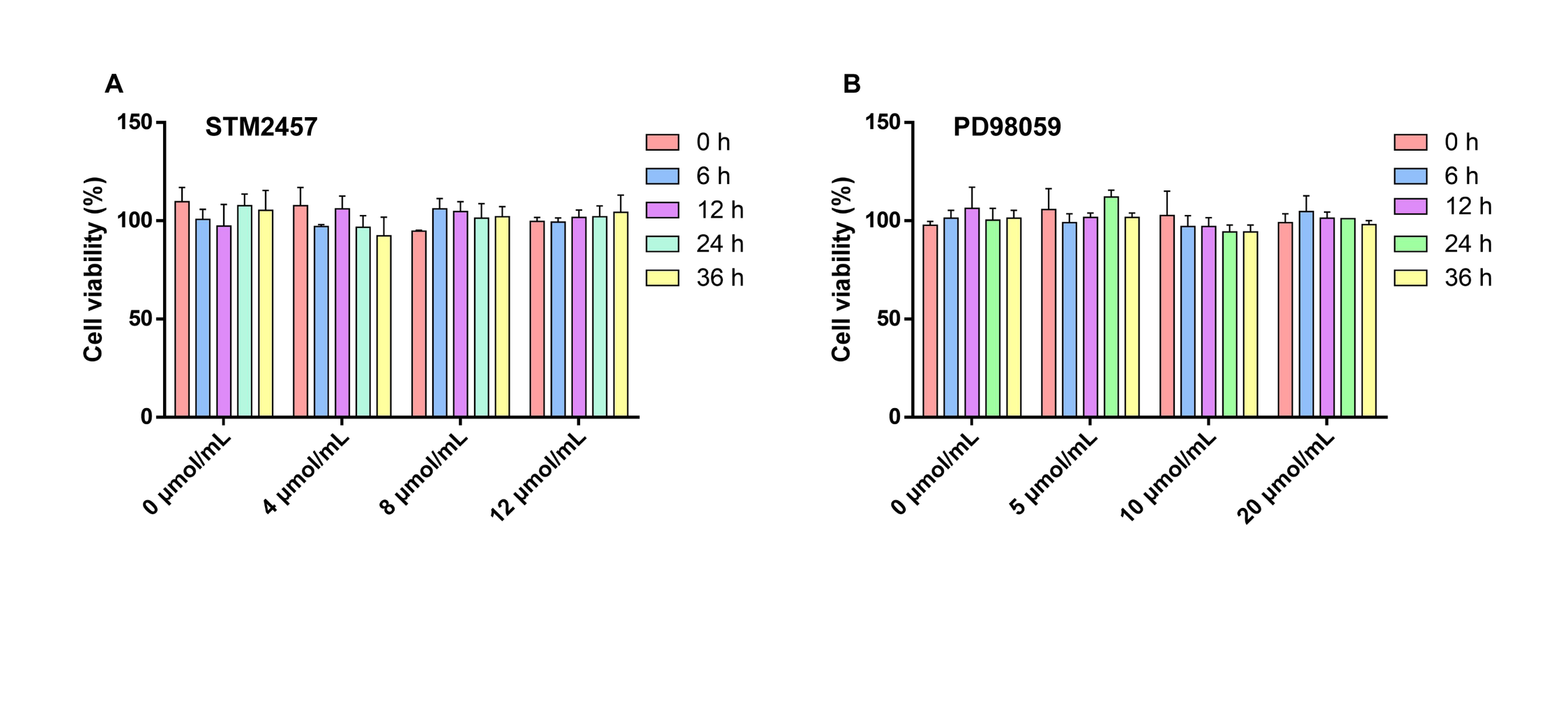

Supplement: S9 Fig — (A) HeLa cells were treated with varying concentrations of STM2457 (0–12 μmol/mL) for 0–36 h. Cell viability was assessed using the CCK-8 assay. (B) HeLa cells were treated with varying concentrations of PD98059 (0–20 μM) for 0–36 h. Cell viability was assessed using the CCK-8 assay. (TIF) [file ppat.1013234.s009.tif]

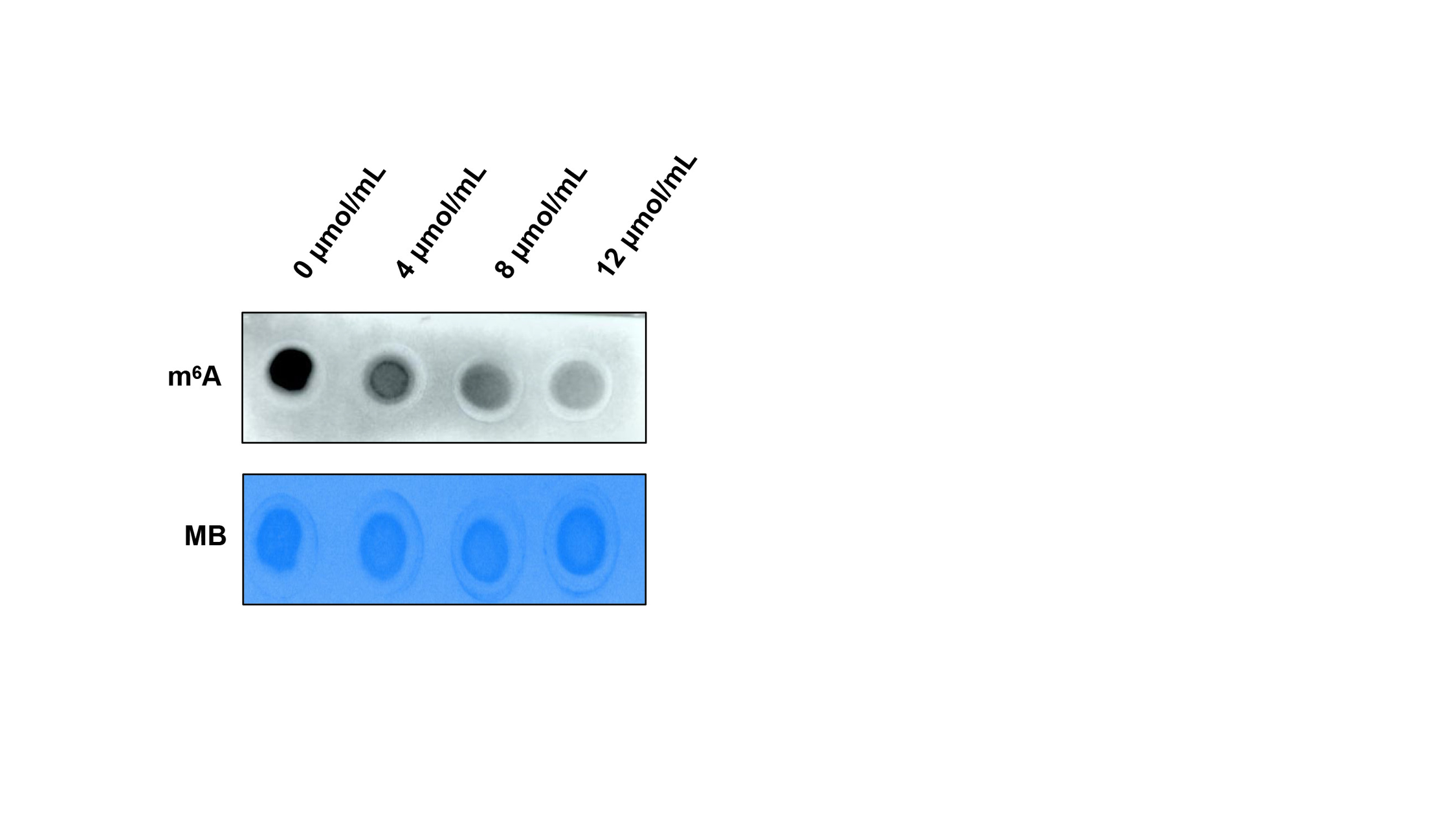

Supplement: S10 Fig — m6A dot blot analysis was conducted to evaluate m6A levels in HeLa cells treated with STM2457 (0–12 μM). (TIF) [file ppat.1013234.s010.tif]
