# Supplementary material for: ERK-METTL3 axis acts as a novel regulator of antiviral innate immunity combating pseudorabies virus infection
Source: PLoS Pathog. 2025 Aug 13;21(8):e1013234. doi: 10.1371/journal.ppat.1013234 (PMC12349697; doi:10.1371/journal.ppat.1013234)
Supplement: S1 Table — (DOCX) [file ppat.1013234.s011.docx]

**S1 Table.** List of primers for plasmid construction and qPCR

| Primer number | Primer name | Sequence 5’-3’ |
| --- | --- | --- |
| 1 | ERK2-F | TTCCAGATTACGCTGAATTCATGGCGGCGGCGGCGGCG |
| 2 | ERK2-R | ATTAAAGATCTGCTAGCTCGAGTTAAGATCTGTATCCTGGCTGGAA |
| 3 | METTL3-F | TCCCCCGGGTAATGTCGGACACGTGGAGC |
| 4 | METTL3-R | TCCGCTAGCTAAATTCTTAGGTTTAGAGATGATACC |
| 5 | PRV gE-F | ATCTGGCTCTGCGTGCTGTG |
| 6 | PRV gE-R | CCGTCGTAGTAGTCCTCGTGC |
| 7 | PRV gB-F | CAAGGCCCACATCTACTACAAGAACGTCATC |
| 8 | PRV gB-R | AGGCGGTCACCTTGTGGTTGTTGC |
| 9 | GAPDH-F | AGCCACATCGCTCAGACAC |
| 10 | GAPDH-R | GCCCAATACGACCAAATCC |
| 11 | shERK2-F | CACCGGACCTCATGGAAACAGATCTCTCGAGAGATCTGTTTCCATG AGGTCCTTTTTTG |
| 12 | shERK2-R | GATCCAAAAAAGGACCTCATGGAAACAGATCTCTCGAGAGATCTGTTTCCATGAGGTCC |
| 13 | ShMETTL3-F | CACCGCCTTAACATTGCCCACTGATCTCGAGATCAGTGGGCAATGTTAAGGCTTTTTTG |
| 14 | ShMETTL3-R | GATCCAAAAAAGCCTTAACATTGCCCACTGATCTCGAGATCAGTGGGCAATGTTAAGGC |
| 15 | sgERK2-1-F | CACCGATCCAGACCATGATCACACA |
| 16 | sgERK2-1-R | AAACTGTGTGATCATGGTCTGGATC |
| 17 | sgERK2-2-F | CACCGCAACCTCTCGTACATCGGCG |
| 18 | sgERK2-2-R | AAACCGCCGATGTACGAGAGGTTGC |
| 19 | sgERK2-3-F | CACCGCCTACTGCCAGAGAACCCTG |
| 20 | sgERK2-3-R | AAACCAGGGTTCTCTGGCAGTAGGC |
| 21 | sgMETTL3-1-F | CACCGATTCTGTGACTATGGAACCA |
| 22 | sgMETTL3-1-R | AAACTGGTTCCATAGTCACAGAATC |
| 23 | sgMETTL3-2-F | CACCGCTTGCTCTTACACAGAGTGT |
| 24 | sgMETTL3-2-R | AAACACACTCTGTGTAAGAGCAAGC |
| 25 | sgMETTL3-3-F | CACCGGAGTTGATTGAGGTAAAGCG |
| 26 | sgMETTL3-3-R | AAACCGCTTTACCTCAATCAACTCC |
| 27 | METTL3-ΔNLS-F | GGGAGGTAGCAGGGACTGTCTCGACTACAGTAGCTGCCTT |
| 28 | METTL3-ΔNLS-R | AAGGCAGCTACTGTAGTCGAGACAGTCCCTGCTACCTCCC |
| 29 | METTL3-1-350-F | GTTCCAGATTACGCTGAATTCATGTCGGACACGTGGAGCTC |
| 30 | METTL3-1-350-R | ATTAAGATCTGCTAGCTCGAGCTAGCTTGGCGTGTGGTCTTTG |
| 31 | METTL3-351-580-F | GTTCCAGATTACGCTGAATTCCAGGAGCTTGCTCTTACACAGAGT |
| 32 | METTL3-351-580-R | ATTAAGATCTGCTAGCTCGAGCTATAAATTCTTAGGTTTAGAGATGATACCAT |
| 33 | METTL3-Δ351-408-F | AAGACCACACGCCAAGCCTGACAGATGATGATGAGATG |
| 34 | METTL3-Δ351-408-R | CATCTCATCATCTGTCAGGCTTGGCGTGTGGTCTT |
| 35 | METTL3-Δ408-465-F | GAACTGCCCTATGGGACCATCATTCGGACAGGCCGT |
| 36 | METTL3-Δ408-465-R | ACGGCCTGTCCGAATGATGGTCCCATAGGGCAGTTC |
| 37 | METTL3-S43 -F | CCAGAGGCAGCATTGGCCCCAACCTTCCGT |
| 38 | METTL3-S43R | ACGGAAGGTTGGGGCCAATGCTGCCTCTGG |
| 39 | METTL3-S50-F | ACCTTCCGTAGTGACGCACCAGTGCCTACT |
| 40 | METTL3-S50-R | AGTAGGCACTGGTGCGTCACTACGGAAGGT |
| 41 | METTL3-S525-F | ATGATTGAAAGACTAGCTCCTGGCACTCGC |
| 42 | METTL3-S525-R | GCGAGTGCCAGGAGCTAGTCTTTCAATCAT |
| 43 | METTL3-S67-F | AGCCCAGCACAGCTGCAGCAGTTCCTGAATT |
| 44 | METTL3-S67-R | AATTCAGGAACTGCTGCAGCTGTGCTGGGCT |
| 45 | ISG15-F | CGCAGATCACCCAGAAGATCG |
| 46 | ISG15-R | TTCGTCGCATTTGTCCACCA |
| 47 | ISG56-F | GCGCTGGGTATGCGATCTC |
| 48 | ISG56-R | CAGCCTGCCTTAGGGGAAG |
| 49 | IFNβ1-F | CAACAAGTGTCTCCTCCAAAT |
| 50 | IFNβ1-R | TCTCCTCAGGGATGTCAAAG |
| 51 | IL-6-F | ACTCACCTCTTCAGAACGAATTG |
| 52 | IL-6-R | CCATCTTTGGAAGGTTCAGGTTG |
| 53 | IL-8-F | CAAGGCTGGTCCATGCTCC |
| 54 | IL-8-R | TGCTATCACTTCCTTTCTGTTGC |
| 55 | sus-sgMETTL3-1-F | AAACCCTGAGTCCTGCTTCCGCC |
| 56 | sus-sgMETTL3-1-R | CACCGGCGGAAGCAGGACTCAGG |
| 57 | sus-sgMETTL3-2-F | CACCGCTGTACCCACTTCTGGTGG |
| 58 | sus-sgMETTL3-2-R | AAACCCACCAGAAGTGGGTACAGC |
| 59 | sus-sgMETTL3-3-F | CACCGCAAGCTCTCTGACCTCTGG |
| 60 | sus-sgMETTL3-3-R | AAACCCAGAGGTCAGAGAGCTTGC |
| 61 | sus-sgERK2-1-F | CACCGCAGTCCTAGAATGCAATGG |
| 62 | sus-sgERK2-1-R | AAACCCATTGCATTCTAGGACTGC |
| 63 | sus-sgERK2-2-F | CACCGGTTTCACACTTTCTCAGG |
| 64 | sus-sgERK2-2-R | AAACCCTGAGAAAGTGTGAAACC |
| 65 | sus-sgERK2-3-F | CACCGCTTCCACACAAAAATAAGG |
| 66 | sus-sgERK2-3-R | AAACCCTTATTTTTGTGTGGAAGC |
